# Novel Immune-Related Ferroptosis Signature in Esophageal Cancer: An Informatics Exploration of Biological Processes Related to the TMEM161B-AS1/hsa-miR-27a-3p/GCH1 Regulatory Network

**DOI:** 10.3389/fgene.2022.829384

**Published:** 2022-02-24

**Authors:** Min Lu, Jiaqi Li, Xin Fan, Fei Xie, Jie Fan, Yuanping Xiong

**Affiliations:** ^1^ Department of Emergency, Shangrao People’s Hospital, Shangrao Hospital Affiliated to Nanchang University, Shangrao, China; ^2^ School of Stomatology, Nanchang University, Nanchang, China; ^3^ Department of Otolaryngology-Head and Neck Surgery, The First Affiliated Hospital of Nanchang University, Nanchang, China; ^4^ Shangrao Municipal Hospital, Shangrao, China

**Keywords:** immune-related ferroptosis, signature, TMEM161B-AS1/ hsa-miR-27a-3p/GCH1 regulatory network, esophageal cancer, clinical value

## Abstract

**Background:** Considering the role of immunity and ferroptosis in the invasion, proliferation and treatment of cancer, it is of interest to construct a model of prognostic-related differential expressed immune-related ferroptosis genes (PR-DE-IRFeGs), and explore the ferroptosis-related biological processes in esophageal cancer (ESCA).

**Methods:** Four ESCA datasets were used to identify three PR-DE-IRFeGs for constructing the prognostic model. Validation of our model was based on analyses of internal and external data sets, and comparisons with past models. With the biological-based enrichment analysis as a guide, exploration for ESCA-related biological processes was undertaken with respect to the immune microenvironment, mutations, competing endogenous RNAs (ceRNA), and copy number variation (CNV). The model’s clinical applicability was measured by nomogram and correlation analysis between risk score and gene expression, and also immune-based and chemotherapeutic sensitivity.

**Results:** Three PR-DE-IRFeGs (DDIT3, SLC2A3, and GCH1), risk factors for prognosis of ESCA patients, were the basis for constructing the prognostic model. Validation of our model shows a meaningful capability for prognosis prediction. Furthermore, many biological functions and pathways related to immunity and ferroptosis were enriched in the high-risk group, and the role of the TMEM161B-AS1/hsa-miR-27a-3p/GCH1 network in ESCA is supported. Also, the KMT2D mutation is associated with our risk score and SLC2A3 expression. Overall, the prognostic model was associated with treatment sensitivity and levels of gene expression.

**Conclusion:** A novel, prognostic model was shown to have high predictive value. Biological processes related to immune functions, KMT2D mutation, CNV and the TMEM161B-AS1/hsa-miR-27a-3p/GCH1 network were involved in ESCA progression.

## Introduction

As the sixth leading cause of death, and the ninth most common cancer in the world, esophageal cancer (ESCA) is mainly composed of two pathological types: esophageal squamous cell carcinoma (ESCC) and esophageal adenocarcinoma (Bray et al., 2018). Squamous cell carcinoma is the most common histological type of ESCA worldwide (Napier et al., 2014). Surgery, the main treatment method for ESCA, is likely to be accompanied by complications such as esophageal obstruction and stenosis (Kofoed et al., 2015). The use of esophageal chemotherapy combined with surgery can optimize the treatment outcome, but drug resistance will lead to chemotherapy failure. With the development and improvement of ESCA treatment strategies, the 5-year survival rate of patients with early ESCA has increased significantly. However, the prognosis of patients with advanced ESCA is still poor (Ferlay et al., 2015). In addition, distant metastasis of cancer will lead to a poor prognosis for patients with ESCA. In recent years, the role of immune checkpoint inhibitors (ICIs) in cancer treatment has brought new hope to ESCA patients.

Previous studies have shown that high expression of PD-L1 in tumors is associated with poor prognosis, while other studies have shown that PD-L1 positive ESCA patients have a higher response rate to immunotherapy (Huang and Fu, 2019; Hong et al., 2020). So, it is inevitable that the heterogeneity of PD-L1 expression will affect the accuracy of prognosis and prediction. Therefore, it is of interest to explore biomarkers that can effectively predict the prognosis of ESCA patients and provide guidance for the best treatment plan.

The immune system plays an irreplaceable role in the occurrence and development of cancer (Gentles et al., 2015), including a significant impact on the effect of radiotherapy. After radiotherapy, the weakened T cell immune function can change the host’s immune response and affect ESCA prognosis and outcome (Hong et al., 2014). In addition, the immune microenvironment composed of immune cells and stromal cells occupies an important position in the progression of tumors (Gajewski et al., 2013). Furthermore, immune infiltrating cells, a crucial factor in the prognosis of tumor cells, are widely used to evaluate the clinical benefits of immunotherapy (Camisaschi et al., 2014). The immunosuppressive cells in the tumor microenvironment can interfere with immune monitoring, leading to tumor immune escape (Zamarron and Chen, 2011). As ICIs-related immunotherapies are widely used in cancer treatment, the predictive value of immune-related genes has also been confirmed by past work (Dine et al., 2017).

Ferroptosis is a cell death pathway driven by iron-dependent lipid peroxidation (Stockwell et al., 2017). Mou et al. (2019) found that LDL-DHA induces cancer cell death through the ferroptosis pathway in liver cancer. In renal cell carcinoma, Miess et al. (2018) found that increased fatty acid metabolism due to β-oxidation leads to lipid peroxidation in renal cell carcinoma and promotes cell ferroptosis. These studies all confirmed the important role of ferroptosis in the progression and prognosis of cancer. Likewise, the potential anti-tumor activity of ferroptosis also shows potential for treatment of metastatic and malignant tumors resistant to traditional therapies. Moreover, activated CD8^+^ T cells can enhance ferroptosis-specific lipid peroxidation in tumor cells (Wang et al., 2019), and which also reflects the cooperative role of immunity and ferroptosis in anti-tumor immunity. However, biological markers constructed based on these two types of genes are rarely reported.

With the in-depth study of the competing endogenous RNA (ceRNA) regulatory network, the interaction mechanism between RNAs has been studied more frequently. The combination of microRNA (miRNA) and mRNA will lead to gene silencing (Salmena et al., 2011). In addition, lncRNA can regulate the expression of target genes by competitively binding with miRNA (Qi et al., 2015). Sequence changes caused by the ceRNA regulatory network play an important role in cell metabolism and the occurrence and development of cancer (Karreth and Pandolfi, 2013). Through ceRNAs analysis, we can further explain how transcripts construct gene expression regulatory networks and explore the mechanism of regulatory genes from a higher scale perspective.

The purpose of this study is to construct a predictive model with excellent performance and that is verifiable by screening the prognostic-related differentially expressed immune-related ferroptosis genes (PR-DE-IRFeGs) in ESCA. We also designed a series of in-depth analyses from the perspective of the tumor immune microenvironment, mutation and ceRNA regulatory axis; and gene copy number variation (CNV) to further explore the potential biological processes closely related to ferroptosis and immunity. In addition, we explored the potential clinical application value of predictive models from multiple aspects, including immunotherapy and chemotherapy. Finally, we construct a nomogram with proven high accuracy to predict the overall survival of ESCA patients. It is hoped that our prediction model can help to further understand the molecular mechanism of ESCA and provide guidance toward the clinical diagnosis and treatment of ESCA.

## Methods

### Data Collection for the Identification of DE-IRFeGs

**FIGURE 1:**
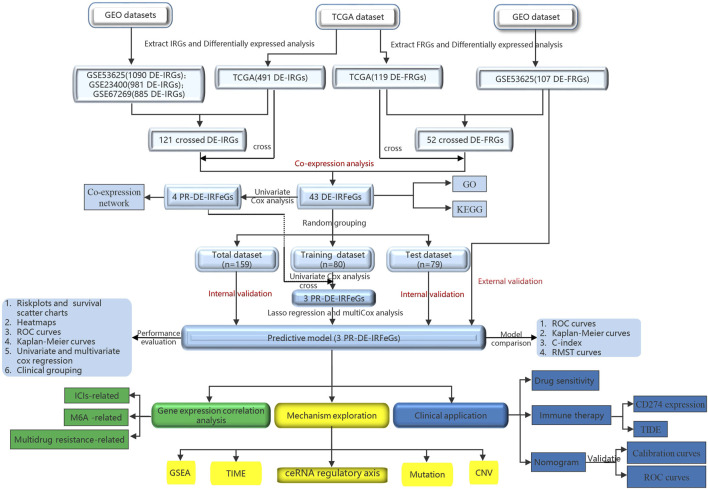
Research diagram of the informatics procedure.

Figure 1 shows a flow chart of the procedure of this study. This study obtained the data of ESCA samples from four public datasets. First, on 1 November 2021, we extracted 171 cases (160 ESCAs and 11 adjacent normal tissues) from The Cancer Genome Atlas database (TCGA, cancergenome.nih.gov) for RNA sequencing and corresponding clinical data. Then, on 2 November 2021, we obtained three external datasets from the Gene Expression Omnibus (GEO) database (https://www.ncbi.nlm.nih.gov/geo/). These are 358 samples from the GSE53625 dataset (179 esophageal squamous cell carcinoma, 179 normal esophagus tissues), and 106 samples from the GSE23400 dataset (53 esophageal squamous cell carcinoma, 53 normal esophagus tissues), and 226 samples (113 esophageal squamous cell carcinoma, 113 normal esophagus tissues) from GSE67269 dataset.

On that date, we also downloaded 2,660 immune-related genes (IRGs) from the ImmPort (www.immport.org/home) and InnateDB (www.innatedb.ca) databases, as well as 259 ferroptosis-related genes (FRGs) from the FerrDb (www.zhounan.org/ferrdb) database. Based on these genes, we extracted the RNA sequencing data of 2,367, 1,540, 1,638, and 1,637 IRGs from the TCGA, GSE53625, GSE23400, and GSE67269 datasets, respectively, as well as the RNA sequencing data of 246, 163, 211, and 211 FRGs from the TCGA, GSE53625, GSE23400, and GSE67269 datasets, respectively.

To analyze the differential expression of IRGs between 160 ESCA tissues and 11 adjacent normal tissues in the TCGA dataset, we set |log2 fold change| (| log2FC |)>1 and false discovery rate (FDR) < 0.05 as filter conditions (R package limma). We also used FDR <0.05 as new filter conditions and analyzed RNA sequencing data of IRGs from GSE53625, GSE23400, and GSE67269 datasets, FRGs from TCGA and GSE53625 datasets to identify the corresponding differentially expressed immune-related genes (DE-IRGs) and DE-FRGs. A fold change of two-fold or greater is considered differential regulation of the protein (Teister et al., 2017). Limited by the restricted number of DE-FRGs and DE-IRGs for sufficient PR-DE-IRFeGs in this differential analysis, we did not use fold change. Finally, we used R package Venn to obtain the common DE-IRGs from TCGA, GSE53625, GSE23400, and GSE67269 datasets, and the common DE-FRGs from TCGA and GSE53625 datasets, respectively. In the process, we only considered the same name of the differentially expressed genes, but did not consider the same direction of the differential expression of these genes.

By co-expression analysis, the threshold was set to the correlation coefficient >0.3 and *p*-value <0.001. The expression values of 121 crossed IRGs, and 52 crossed FRGs extracted from the expression matrix of TCGA were used to filter differentially expressed immune-related ferroptosis genes (DE-IRFeGs).

### GO and KEGG Enrichment Analysis Based on DE-IRFeGs

To show that the functions and pathways of DE-IRFeGs have been enriched, we searched the databases of Kyoto Encyclopedia of Genes and Genomes (KEGG) and Gene Ontology (GO) for these DE-IRFeGs (R package org.Hs.eg.db). In addition to histograms, bubble charts were also used to display significantly rich functions and pathways.

### Recognition of PR-DE-IRFeGs and Construction of the Predictive Model

We extracted the same samples with complete overall survival (OS) and mRNA expression data from the TCGA and GSE53625 datasets. To obtain DE-IRFeGs with prognostic values from the TCGA, we performed univariate Cox analysis with a cutoff value of *p* < 0.05.

To elucidate the differential expression of 4 PR-DE-IRFeGs in ESCA, we used the R package ConsensusClusterPlus (1,000 iterations and 80% resampling rate) to classify ESCA patients into different subtypes. A heat map was used to demonstrate the differences of clinicopathological features and 4 PR-DE-IRFeGs expression between the two subtypes. We also performed Kaplan-Meier survival analysis on the PR-DE-IRFeGs to explore the relationship between their expression and OS. In addition, we also mapped the co-expression network between these PR-DE-IRFeGs and the corresponding DE-IRGs. 159 samples with complete OS data and PR-DE-IRFeGs mRNA expression values were randomly matched to the training dataset (*n* = 80) and test dataset (*n* = 79) on average. To verify that randomization did not cause a deviation in the distribution of clinical traits, we used a chi-square test to compare the differences in clinical characteristics between the training and the test datasets.

Similarly, we extracted PR-DE-IRFeGs shared by the PR-DE-IRFeGs obtained from the training dataset and the PR-DE-IRFeGs obtained from the TCGA total dataset. Lasso regression analysis can screen out highly relevant crossed PR-DE-IRFeGs in the training set, thereby minimizing the risk of overfitting of screening features and achieving the purpose of accurately predicting the clinical prognosis of patients. Then, we determined the penalty parameter (*λ*) through the minimum 10-fold cross-validation and selected the optimal penalty parameter (*λ*) from it to construct a multiCox regression model (predictive model) based on 3 PR-DE-IRFeGs. Finally, we applied the coefficients obtained by the lasso and multiCox regression algorithm to the following risk scoring equation:

Riskscore=∑(PR−DE−IRFeGs exp⁡ression values×corresponding coefficient)

### Validation of Predictive Model

We used the training dataset, test dataset, and total dataset from the TCGA database and the external dataset GSE53625 to evaluate and verify the accuracy of the established predictive model in predicting prognosis. All samples in the TCGA and GSE53625 datasets were assigned risk scores, and ESCA patients were divided into high-risk and low-risk groups using the median of the risk scores as a cutoff score. After obtaining the risk score, we used R to visualize each sample’s specific risk score and survival status. We created a Kaplan-Meier curve to clarify the correlation between risk score and patient survival index, and visualized the risk plot, survival status, and heatmap of four datasets through related R packages to further verify the accuracy of the predictive model. According to the patient’s risk score and overall survival, the Receiver Operating Characteristic (ROC) curve was drawn. The R package timeROC was used to predict ESCA patient survival for 1, 2, and 3-years. The area under the curve (AUC) value represents the accuracy of prediction. Univariate and multivariate Cox regression was used to verify the independence of the predictive model and analyze whether the risk score could still be used as an independent predictor for the patient’s survival under the case of multifactorial clinical characteristics (age, gender, T stage, N stage, and clinical stage).

### Comparing Prediction Performance With Other Models

Song et al. (2021) and Tang et al. (2021), respectively, screened 7 ferroptosis-related genes and 4 autophagy-related genes to construct models to predict the prognosis of patients with ESCA. We extracted the mRNA expression data of the corresponding genes of each model from the TCGA ESCA dataset to construct a multiCox regression model and calculated the corresponding risk score for each sample. Similarly, the samples were divided into high-risk and low-risk groups based on the median risk scores of all examples in each model. The ROC plots based on the risk scores of the three model samples were used to compare the performance of the models in predicting prognosis.

Similarly, the Kaplan–Meier survival plot were used to compare the ability of the three models to distinguish prognosis. Thus, we tried to compare the prognostic performance of the 7-gene combination model, 4-gene combination model and our 3-gene combination model based on the concordance index (C-index) calculated based on the mRNA expression levels of the 7 genes (ALOX12, ALOX12B, ANGPTL7, DRD4, MAPK9, SLC38A1, and ZNF419) that Song et al. (2021) introduced, the 4 genes (SQSTM1, BIRC5, NRG3, and CXCR4) that Tang et al. (2021) introduced, and the 3 genes our study presented. Higher C-index implied better prognostic performance (Schröder et al., 2011). The R package survcomp was used to calculate and compare the C-indexes between the 3 prognostic combinations (Schröder et al., 2011). In addition, the restricted mean survival time (RMST) curve was also used to evaluate the performance of each model and compare the differences among them (Zheng et al., 2021).

### Stratified Analysis of Predictive Model

The stratified analysis tested whether the predictive model was highly accurate in different clinicopathological feature groups. First, heat maps were used to display the clinical features of all ESCA samples in the high-risk and low-risk groups. According to different clinical parameters, including survival status (Alive and Deceased), gender (female and male), tumor stage (I-II and III-IV), T stage (T0-T4), M stage (M0-M1), N stage (N0-N3), divide the entire TCGA concentration into patients for the subgroup. Box plots show the differences of riskscore in different subgroups. Kaplan-Meier analysis and log-rank test were performed to compare the survival differences between the high-risk and the low-risk groups in each subgroup.

### Biological-Based Enrichment Analysis and Immune Infiltration Assessment

To explore the immune biological functions and pathways involved in different risk groups, we used an R package for GO biological function enrichment analysis and KEGG pathway enrichment analysis based on differentially expressed genes between different risk groups. The R package cluster profile and gene sets “c2.cp.kegg.v7.4.symbols.gmt” and “c5.go.v7.4.symbols.gmt” were used in this process. Considering the enrichment of a large number of immune-related functions and pathways in the high-risk group, the next step was to use the R package estimate to calculate the immune and stromal cell fractions of each sample. In addition, we also compared the differences in the immune and stromal cell fractions of patients between different risk groups. Based on the single-sample gene set enrichment analysis (ssGSEA) of the R packages GSEAbase and gsva, we obtained 16 immune cells and 13 immune function scores to estimate each abundance of infiltrating immune cells and functions in each sample. Firstly, based on the predictive model, the difference analysis of immune cells and functions between different risk groups was carried out, and a box plot was drawn. Heat maps show the distribution differences of 16 immune cells and 13 immune functions in each sample with different risk scores. In addition, correlation analysis was conducted to evaluate the relationship between each patient’s immune cell/function score and risk score. Finally, the differences of 16 kinds of immune cells and 13 kinds of immune functions between the high and low-risk groups were compared.

To assess the composition of different immune cell types in ESCA, we also used the Cibersort deconvolution algorithm to obtain matrix data for the proportion of 22 immune cells per tumor sample from RNA-sequencing data. We further visualized matrix based data filtered by *p* < 0.05 with the bar chart. We also performed correlation analysis between different immune cells and visualized the corresponding results in the correlation matrix plot of immune cells.

### Prediction and Verification of ceRNA Regulatory Network

For the purpose of determining the interaction between lncRNAs, miRNA, and mRNAs, we combined the data of lncRNAs and mRNAs with miRNA data to construct the lncRNA-miRNA-mRNA regulatory network, and further explore the putative mechanism of ESCA progress. We select GCH1 in the model to predict and verify the complete ceRNA regulatory axis. In order to further verify the universal differential expression of GCH1 between the cancer group and the standard group, we downloaded the RNA sequencing data of 33 human cancers from the UCSC Xena (https://xena.ucsc.edu/) database. These annotated RNA sequencing data were used to differentiate GCH1 expression between cancer and normal tissues. GEPIA (gepia.cancer-pku.cn) is a tool for cancer and standard gene expression profiling and interactive analysis (Tang et al., 2017). This website was used to verify further the difference in survival based on GCH1 expression in ESCA. Next, the miRNA expression data of 185 ESCA tissues and 13 normal tissues adjacent to cancer were obtained from TCGA. After annotating with the mature miRNA annotation file downloaded from mirbase (https://www.mirbase.org/), we received the miRNA expression matrix of these 198 samples. After the prediction by multiple target gene prediction programs, including PITA, RNA22, minimap, microT, miRanda, PicTar and TargetScan in StarBase (starbase.sysu.edu.cn), miRNAs in the upstream binding of GCH1, appeared more than twice, were considered candidate miRNAs for GCH1. We use Cytoscape (v3.8.2) to map the co-expression network of miRNA and GCH1. The R packages ggExtra and reshape2 were employed to obtain the correlation between GCH1 expression level and upstream binding miRNA. The differential expression of miRNAs (correlation coefficient *t* < −0.34, *p* < 0.001) between tumor and normal tissues is dependent on the difference analysis (|log2FC|) > 1, *p* < 0.05). The Kaplan-Meier plotter was used to draw survival plots between the subgroups with high and low miRNA expression. Only the analysis result of hsa-miR-27a-3p was statistically significant and used for subsequent analysis. StarBase (v2.0) was also used to predict candidate lncRNAs that binds to hsa-miR-27a-3p. We reused Cytoscape (v3.7.2) to map the co-expression network of lncRNAs and hsa-miR-27a-3p. Similarly, the correlation between lncRNAs and hsa-miR-27a-3p expression (correlation coefficient *t* < −0.31, *p* < 0.001) and the correlation between lncRNAs and GCH1 expression, as well as the difference (|log2FC|) > 1, *p* < 0.05) and survival analysis of lncRNAs (*p* < 0.05), were also analyzed. Only the analysis result of TMEM161B-AS1 was statistically significant. Finally, reran Cytoscape (v3.7.2) to draw the ceRNA regulatory network composed of hsa-miR-27a-3p, TMEM161B-AS1, and GCH1.

### Analysis of the Correlation Between PR-DE-IRFeGs and Mutation Field

In order to analyze the correlation between mutations and predictive model, we downloaded the somatic gene mutation data and corresponding clinical data of ESCA samples from the TCGA dataset. After using VarScan to detect the MAF files of somatic mutations in ESCA samples, the R package GenVisR was used to visualize the 30 most frequently mutated genes in the high-risk and low-risk groups. The waterfall diagram shows the mutation in the 43 DE-IRFeGs. Tumor mutation burden (TMB) is the number of mutation bases per million bases calculated based on the somatic mutation data of each tumor (Liu C. et al., 2021). We calculated each patient’s TMB using perl. We explored the correlation between TMB and risk score. In addition, we compared the difference in TMB between high and low-risk groups. The Kaplan-Meier survival curve was used to compare the survival difference between the high TMB and low TMB groups. According to the mutation status of KMT2D/MUC16, TCGA samples were divided into wild group and mutation group. The difference between the risk scores between KMT2D/MUC16 mutation and the wild group was compared. In addition, we also analyzed the relationship between KMT2D/MUC16 mutation and the three PR-DE-IRFeGs. To explore the relationship between KMT2D/MUC16 mutations and the prognosis of ESCA relationship, a Kaplan-Meier survival curve analysis was used to compare the wild and mutant groups’ OS differences. Pan-cancer analysis was used to compare the expression differences of KMT2D and MUC16 in cancer tissues and normal tissues of 33 cancer patients. The differences in the expression of KMT2D and MUC16 were found in different tumor types.

### CNV Analysis

CNV data of TCGA ESCA patients were downloaded from the UCSC Xena (https://xena.ucsc.edu/) database On 8 November 2021. The CNV data of 43 DE-IRFeGs in 185 ESCA samples were used in our analysis. After statistics of the CNV frequency of these genes, the corresponding results were visualized. The CNV changes of these 43 DE-IRFeGs on the chromosome were also pictured in the circle diagram, which can well reflect the corresponding position of the gene on the chromosome. We divided all samples into single deletion, normal, and single gain copy number groups based on the change in copy number of 3 PR-DE-IRFeGs in the model, respectively. The Kruskal-Wallis test was used to compare the expression differences of the corresponding PR-DE-IRFeGs among the three groups. In addition, Kaplan–Meier survival plot were used to compare the survival differences of the three groups of samples.

### Correlation Analysis Between Predictive Model and ICIs-Related, m6A-Related and Multidrug Resistance-Related Genes

In view of the fact that the expression levels of ICIs (ICIs)-related genes may be related to the clinical results of immune checkpoint inhibitor blockade treatment, we applied a spearman correlation analysis to explore the correlation between the risk score and the expression of ICIs-related genes. In addition, to verify the accuracy of the correlation results, we also compared the differences in ICIs-related gene expression between samples in the high and low-risk groups. The same method was applied to explore the correlation between the expression of N6-methyladenosine (m6A)-related genes/multidrug resistance-related genes and risk score. The R packages ggplot2 and reshape2 were used in this analysis.

### Clinical Treatment Application of 3 PR-DE-IRFeGs Used to Construct a Predictive Model

Studies have shown that the gene expression levels of critical targets for immune checkpoint blockade may be closely related to the clinical effects of ICIs (Hodi et al., 2010). We selected programmed death-ligand 1 (PD-L1 or CD274) that can be used to predict the effect of immunotherapy for further analysis. The correlation between expression of CD274 and risk score/3 PR-DE-IRFeGs genes were shown by circle diagram. Application of Tumor Immune Dysfunction and Exclusion (TIDE) algorithm and Microsatellite Instability (MSI) could be used to predict the potential response to immune checkpoint blockers (ICB) treatment (Jiang et al., 2018; Lin et al., 2020). We also used the circle graph to show the correlation between TIDE, MSI, Dysfunction, Exclusion, and risk score/3 PR-DE-IRFeGs through Spearman correlation analysis. We used the R package pRophetic to predict the half-maximal inhibitory concentration (IC50) of the three chemotherapeutics as recommended by the National Comprehensive Cancer Network (NCCN) guidelines for treating ESCA patients from the total dataset of samples. Used the cell line expression data in the Cancer Drug Sensitivity Genomics (GDSC) database and the RNA sequencing transcriptome data in the TCGA database to construct a regression model to predict the IC50 of the drug in the R package (Geeleher et al., 2014). We performed Spearman correlation analysis to evaluate the correlation between the IC50 of the three chemotherapeutic drugs and the risk score. We also used the same method to explore further the correlation between the expression of the three PR-DE-IRFeGs and the IC50 of these three drugs. Finally, we compared the IC50 difference between the high-risk group and the low-risk group.

### Construction and Verification of Forecast Nomogram

To create a clinically applicable quantitative tool to predict 1, 2, and 3-year OS of ESCA patients and monitor the prognosis of patients, we combined N and M staging and risk group to generate a nomogram to predict ESCA patients’ survival probability. The nomogram was constructed by using the R package rms. We drew the 1, 2, and 3-year ROC plot of the training dataset, test dataset, and total dataset of the TCGA database. For the purpose of verifying the accuracy of the nomogram, we also used the calibration curve to evaluate the accuracy of the nomogram in survival prediction. In the calibration curve, if the predictive value is more consistent with the actual value, it means that the prediction accuracy of the nomogram is higher.

### Statistical Analysis

In order to compare the differences in risk scores between different subgroups of these clinical features, a chi-square test was used. Next, we used Student’s t-test or Wilcoxon signed-rank test to compare the difference between continuous variables and the chi-square test or Fisher’s exact test to compare the difference between categorical variables. Univariate cox regression analysis was used to identify PR-DE-IRFeGs. lasso regression and mutiCox regression are used to screen PR-DE-IRFeGs to build predictive model. Kaplan-Meier analysis and log-rank test was used to compare OS differences between different subgroups. The univariate and multivariate Cox analysis based on each clinical feature and risk score were used to verify the independent prognostic value of the risk score. Spearman or Pearson correlation analysis was used to analyze the correlation between variables. We used the R programming language (version 4.0.3), Perl, and Cytoscape (version 3.8.2) to run these analyses.

## Results

### Identification of DE-IRFeGs

**FIGURE 2:**
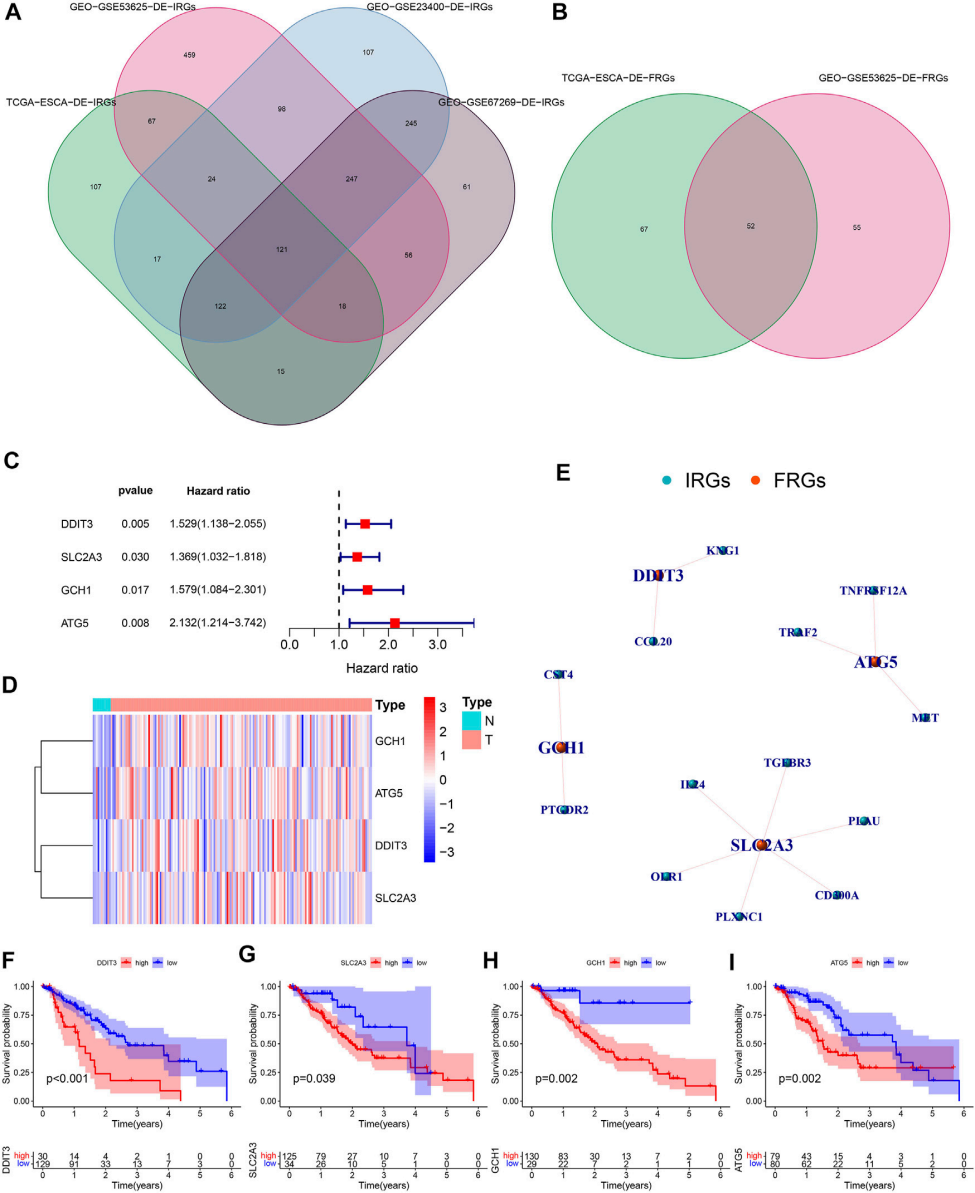
Recognition of PR-DE-IRFeGs. **(A–B)** Genes shared among database samples of DE-IRGs and DE-FRGs. The database samples refer to TCGA and GEO. **(C)** Forest plot of univariate Cox regression analysis of 4 PR-DE-IRFeGs. Hazard ratio is obtained by running single factor and multi-factor cox regression. **(D)** The heat map shows the expression status of 4 PR-DE-IRFeGs in tumor and standard cases. **(E)** The co-expression network between 4 PR-DE-IRFeGs and the corresponding DE-IRGs. **(F–I)** Survival differences between the high and low expression groups that correspond to the four PR-DE-IRFeGs.

We quantified 491 DE-IRGs (of which 103 genes were down-regulated, and 388 genes were up-regulated) in the TCGA dataset (Supplementary Figure S1), 1090 DE-IRGs (of which 598 genes were up-regulated and 492 genes were down-regulated) in the GSE53625 dataset (Supplementary Figure S2), and 981 DE-IRGs (of which 545 genes were down-regulated and 436 genes were up-regulated) in the GSE23400 dataset (Supplementary Figure S3), and 885 DE-IRGs (of which 440 genes were down-regulated and 445 genes were up-regulated) from the GSE67269 dataset (Supplementary Figure S4). Finally, by extracting common DE-IRGs of the 4 datasets, we obtained 121 shared DE-IRGs (Figure 2A). Likewise, we also identified 119 DE-FRGs (of which 24 genes were down-regulated and 95 genes were up-regulated) in the TCGA dataset (Supplementary Figure S5) and 107 DE-FRGs (of which 64 genes were up-regulated and 43 genes were down-regulated) in the GSE53625 dataset (Supplementary Figure S6). Similarly, by intersecting the DE-FRGs of the 2 datasets, we obtained 52 crossed DE-FRGs (Figure 2B). We identified 43 DE-IRFeGs under the condition of performing the co-expression analysis between RNA sequencing data of IRGs and FRGs. Supplementary Table S1 shows the correlation results among IRGs and 43 DE-IRFeGs.

### GO and KEGG Enrichment Analysis Based on DE-IRFeGs

Studies have reported that hypoxia can protect tumor cells from the effects of ferroptosis inducers, and in the case of hypoxia, iron output will increase, and stable ferritin input will decrease, indicating that hypoxia and ferroptosis are closely related (Stockwell et al., 2017). Surprisingly, our DE-IRFeGs genes enriched with biological processes (BPs) are almost all related to hypoxia-related reactions, such as response to oxidative stress, cellular response to oxidative stress, response to oxygen levels, response to hypoxia, response to decreased oxygen level, and reactive oxygen metabolism process (Supplementary Figure S7A). KEGG pathway analysis revealed that these genes are mainly enriched in autophagy, mitochondrial autotropism, HIF-1 signaling pathway, NOD-like receptor signaling pathway, chemical carcinogenesis-reactive oxygen species (Supplementary Figure S7B), and which are related to hypoxia and autophagy, also closely related, indicating that the DE-IRFeGs gene we screened is closely related to ferroptosis.

### Recognition of PR-DE-IRFeGs and Construction of Predictive Model

By integrating the mRNA expression and clinical data of ESCA patients, we obtained 159 samples in the TCGA dataset and 179 samples in the GSE53625 dataset, respectively. Their clinical characteristics were shown in Table 1. 4 PR-DE-IRFeGs (DDIT3, SLC2A3, GCH1, and ATG5) were screened out by univariate cox analysis (Figure 2C). All PR-DE-IRFeGs (HR > 1) were identified as risk factors (Figure 2C).

**TABLE 1:** Sample sizes by clinical characteristic in the four data sets.

Features	Type	Total dataset	Test dataset	Training dataset	P	GSE53625 dataset
Age (years)	< = 60	-	-	-	**-**	99(55.3%)
	>60	-	-	-		80(44.7%)
Futime (days)	< = 730	123(77.36%)	61(77.22%)	62(77.5%)	1	71(39.7%)
	>730	36(22.64%)	18(22.78%)	18(22.5%)		108(60.3%)
Fustat	Alive	96(60.38%)	43(54.43%)	53(66.25%)	0.1734	73(40.8%)
	Deceased	63(39.62%)	36(45.57%)	27(33.75%)		106(59.2%)
Gender	Female	23(14.47%)	11(13.92%)	12(15%)	1	33(18.4%)
	Male	136(85.53%)	68(86.08%)	68(85%)		146(81.6%)
Stage	I	16(10.06%)	6(7.59%)	10(12.5%)	0.3795	10(5.6%)
	II	68(42.77%)	30(37.97%)	38(47.5%)		77(43.0%)
	III	48(30.19%)	27(34.18%)	21(26.25%)		92(51.4%)
	IV	8(5.03%)	5(6.33%)	3(3.75%)		0(0.0%)
	Unknown	19(11.95%)	11(13.92%)	8(10%)		0(0.0%)
T	T0	1(0.63%)	1(1.27%)	0(0%)	0.707	0(0.0%)
	T1	27(16.98%)	14(17.72%)	13(16.25%)		12(6.7%)
	T2	37(23.27%)	19(24.05%)	18(22.5%)		27(15.1%)
	T3	75(47.17%)	36(45.57%)	39(48.75%)		110(61.4%)
	T4	4(2.52%)	1(1.27%)	3(3.75%)		30(16.8%)
	Unknown	15(9.43%)	8(10.13%)	7(8.75%)		0(0.0%)
M	M0	119(74.84%)	59(74.68%)	60(75%)	0.7322	-
	M1	8(5.03%)	5(6.33%)	3(3.75%)		-
	Unknown	32(20.13%)	15(18.99%)	17(21.25%)		-
N	N0	65(40.88%)	25(31.65%)	40(50%)	0.0645	83(46.4%)
	N1	62(38.99%)	35(44.3%)	27(33.75%)		62(34.6%)
	N2	9(5.66%)	7(8.86%)	2(2.5%)		22(12.3%)
	N3	6(3.77%)	3(3.8%)	3(3.75%)		12(6.7%)
	Unknown	17(10.69%)	9(11.39%)	8(10%)		0(%)

Futime represents overall survival; Fustat represents survival state; P represents *p* value; Stage represents clinical stages.

The heat map in Figure 2D showed the expression of these genes. The similarity by the expression levels of the 4 PR-DE-IRFeGs and the proportion of ambiguous clustering determined that *k* = 2 qualitatively showed an ideal clustering pattern (Supplementary Figures S8A–C). All ESCA patients were divided into two subtypes, cluster 1 (*n* = 99) and cluster2 (*n* = 60) (Supplementary Figure S8D). The expression of DDIT3 and SLC2A3 was higher in cluster 2 than in cluster 1 (Supplementary Figure S8E).

In addition, better Overall Survival (OS) was observed in the low expression of these PR-DE-IRFeGs by the Kaplan-Meier survival in Figures 2F–I (*p <* 0.05). Figure 2E shows the co-expression relationship between each PR-DE-IRFeGs and the corresponding DE-IRGs. Table 1 also shows no significant difference in all clinical traits between the training dataset and the test dataset (*p* > 0.05), and which shows that the randomization did not induce bias in the distribution of clinical characteristics data.

We obtained 3 common PR-DE-IRFeGs (DDIT3, SLC2A3 and GCH1) in the training dataset and in the total dataset. Next, 3 PR-DE-IRFeGs, namely DDIT3, SLC2A3, and GCH1, based on the optimal value of λ, were determined by lasso regression analysis and used to construct the multiCox regression model. We calculated the risk score of each sample of 3 datasets in TCGA and the GSE53625 dataset according to the risk score calculation formula: Risk score=∑DDIT3 expression values×0.3605+SLC2A3 expression values×0.3646+GCH1 expression values×0.4714 . Then, based on the median value of the risk scores of all samples in each dataset, the samples were divided into high-risk and low-risk groups.

### Validation of Predictive Model

**FIGURE 3:**
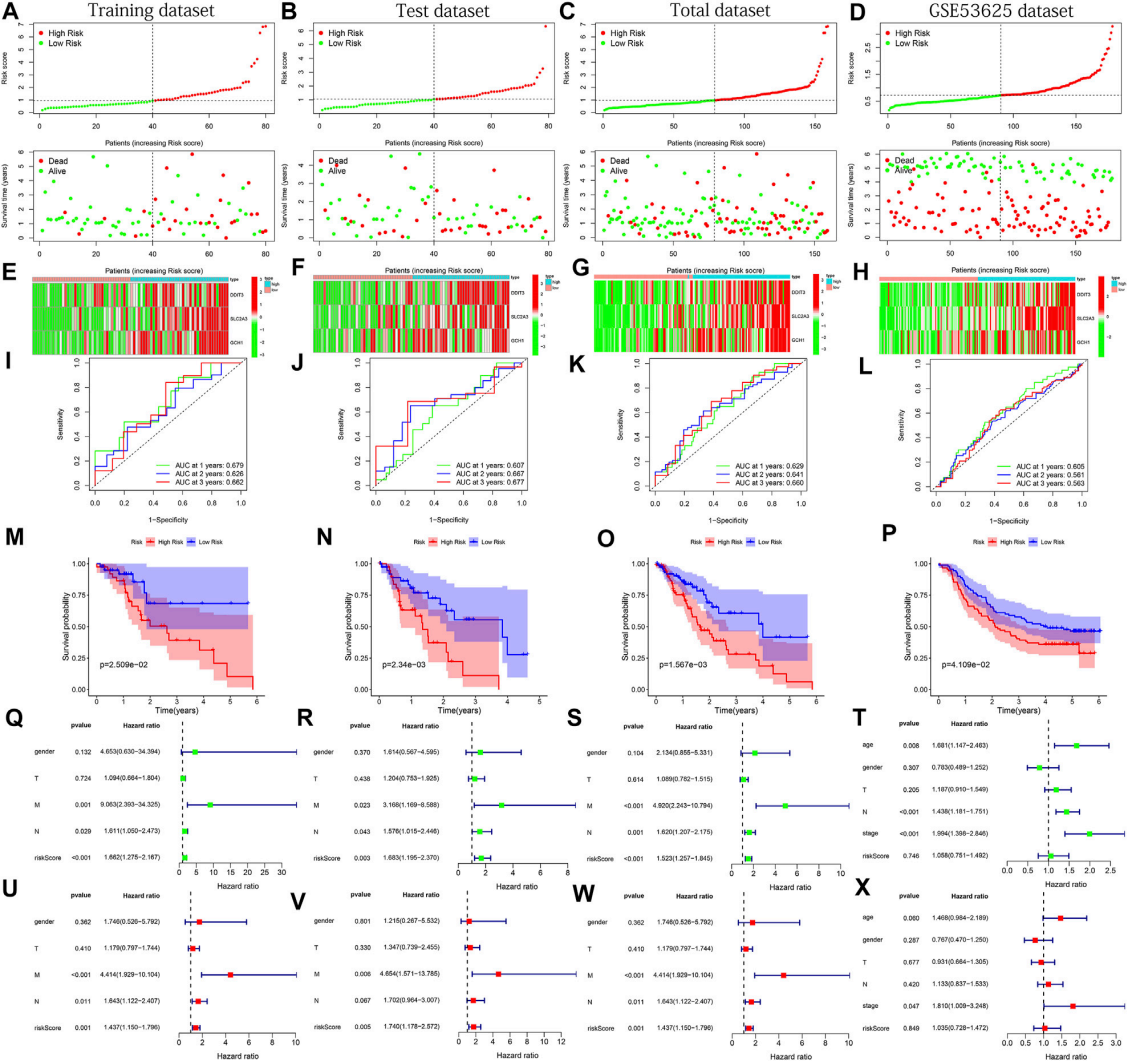
The results of various methods to verify the performance of the model based on the training, test, total and GSE53625 datasets. **(A–D)** Risk score and survival time plots. **(E–H)** Expression heat map of 3 PR-DE-IRFeGs. **(I–L)** 1-, 2-, and 3-year ROC plot **(M–P)** Kaplan-Meier survival plot. **(Q–T)** Forest plots for univariate Cox regression. **(U–X)** Forest plots for multivariate Cox regression.

To verify the applicability and prognostic value of our predictive model based on the TCGA training dataset, we used the training dataset, test dataset, total dataset of the TCGA database and the external dataset GSE53625 for testing. The median of the risk score was used as the cutoff value to divide the four sets of patients into high- and low-risk groups, respectively. The risk curve graph and survival state graph show the distribution of risk scores and the overall survival of the four sets of samples (Figures 3A–D). The heat map shows the distribution of the three genes screened for the predictive model between the high- and low-risk groups (Figures 3E–H). The results show that these three genes have differences between the high and low-risk tissues. Next, we used the ROC curve to check the prediction performance of the model for 1, 2, and 3 years of OS. The results in Figures 3I–K show that the AUC of the training dataset, test dataset, and total dataset is greater in significance than 0.6 (most AUC values > 0.65).

In the external dataset, The AUC of GSE53625 is also greater than 0.5 (Figure 3L). In addition, the results of the Kaplan-Meier plot show that patients with high-risk scores had a lower survival probability than the lower-risk group (Figures 3M–P), verifying that the prognosis of the high-risk group was worse than that of the low-risk group. Finally, we performed univariate and multivariate Cox regression analysis to check whether the risk score is an independent prognostic factor for ESCA patients. We analyzed the association between OS and clinical characteristics (including age, sex, T stage, M stage, N stage, and risk score) of ESCA patients in the training dataset, test dataset, total dataset, and external GSE53625 dataset. After adjusting for other clinical confounding factors, multivariate Cox regression analysis still determined risk score as the independent predictor of each group of OS (Figures 3Q–S, U–W) across 3 TCGA datasets. Unfortunately, similar results were not found in the GSE53625 dataset (Figure 3T,X). In general, our predictive model has good performance for ESCA survival prediction.

### Comparing Prediction Performance With Other Models

**FIGURE 4:**
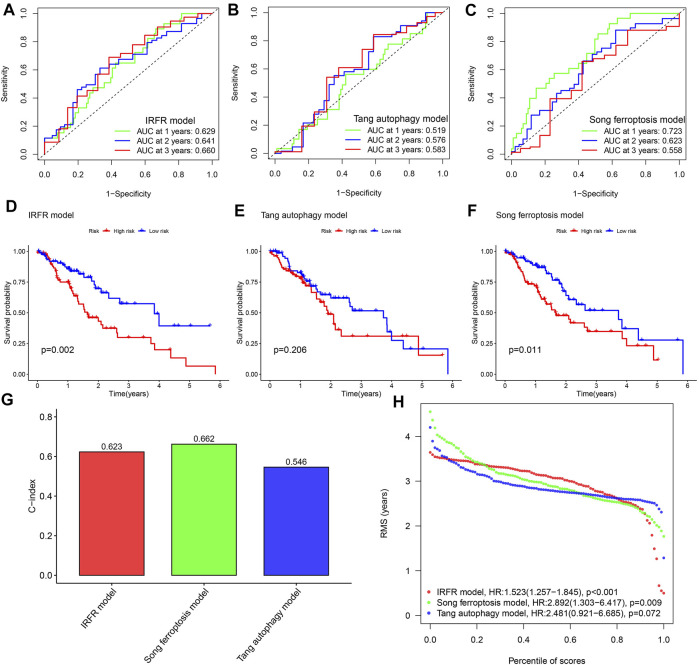
Performance comparison among different models. **(A–C)** The 1, 2, and 3- years ROC curves for three models. **(D–F)** Kaplan-Meier survival curves for three models. **(G)** Comparison of C-index of the three models. **(H)** Comparison of RMST plot of the three models. IRFR model represents the model we built using three PR-DE-IRFeGs. Tang autophagy model represents a model constructed by Tang et al. (2021) using 4 autophagy-related genes. The Song ferroptosis model represents a model constructed by Song et al. (2021) using 7 ferroptosis-related genes.

By comparison, the AUC of our model has observed the highest AUC value in almost all years among the three models (Figures 4A–C), and which means that our model has the best performance in predicting the prognosis of ESCA patients. The Kaplan–Meier survival plot also confirmed that our model with the lowest *p*-value has the best ability to distinguish prognosis (Figures 4D–F). In addition, through the comparison of the c-indexes, we also observed that our model performed better than the model of Tang et al. (2021) (Figure 4G). And our model has also been marked to have the highest RMST curve in 3 models (Figure 4H).

### Stratified Analysis of Predictive Model

**FIGURE 5:**
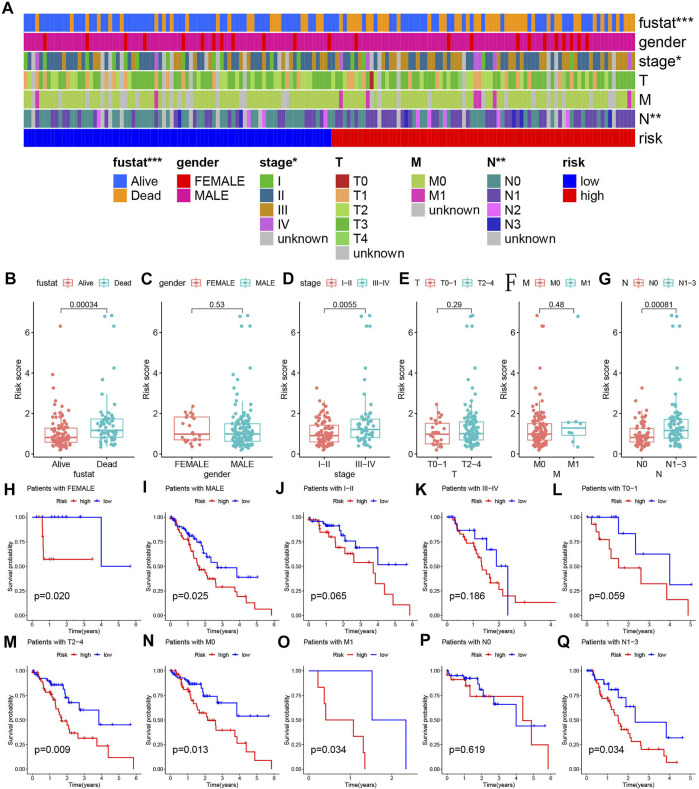
Detailed analyses of clinical data. **(A)** The distribution of clinical characteristics and risk for each data sample. **(B–G)** Differences in risk scores of patients with different clinical characteristics. **(H–Q)** Kaplan-Meier survival plots for different groups by clinical feature.

Since clinical features such as risk score and TNM staging are independent prognostic factors of OS in separate data sets, we used stratified analysis to explore whether the predictive model can effectively predict ESCA patient’s OS with different clinicopathological characteristics. The heat map shows an overview of the clinical features of each patient (Figure 5A). When analyzing the relationship between risk scores and various clinical characteristics, we made several findings of interest. Survival status, stage, and N stage were highly correlated with the risk score (Figures 5B,D,G, *p* < 0.001). In other clinical features, no significant results were found (Figures 5C,E,F, *p* > 0.05). In addition, we implemented the Kaplan-Meier survival curve to verify the predictive value of the model in different clinical parameter subgroups. We found that the predictive model also has good OS predictive performance in each subgroup with different clinical characteristics, except for stage I-II staging, stage III-IV staging, T0-1 staging, and N0 staging (*p*＜0.05). In addition to the N0 staging subgroup, patients in the low-risk group of other subgroups have a better OS (Figures 5H–Q).

### Enrichment Analysis and Immune Infiltration Assessment

**FIGURE 6:**
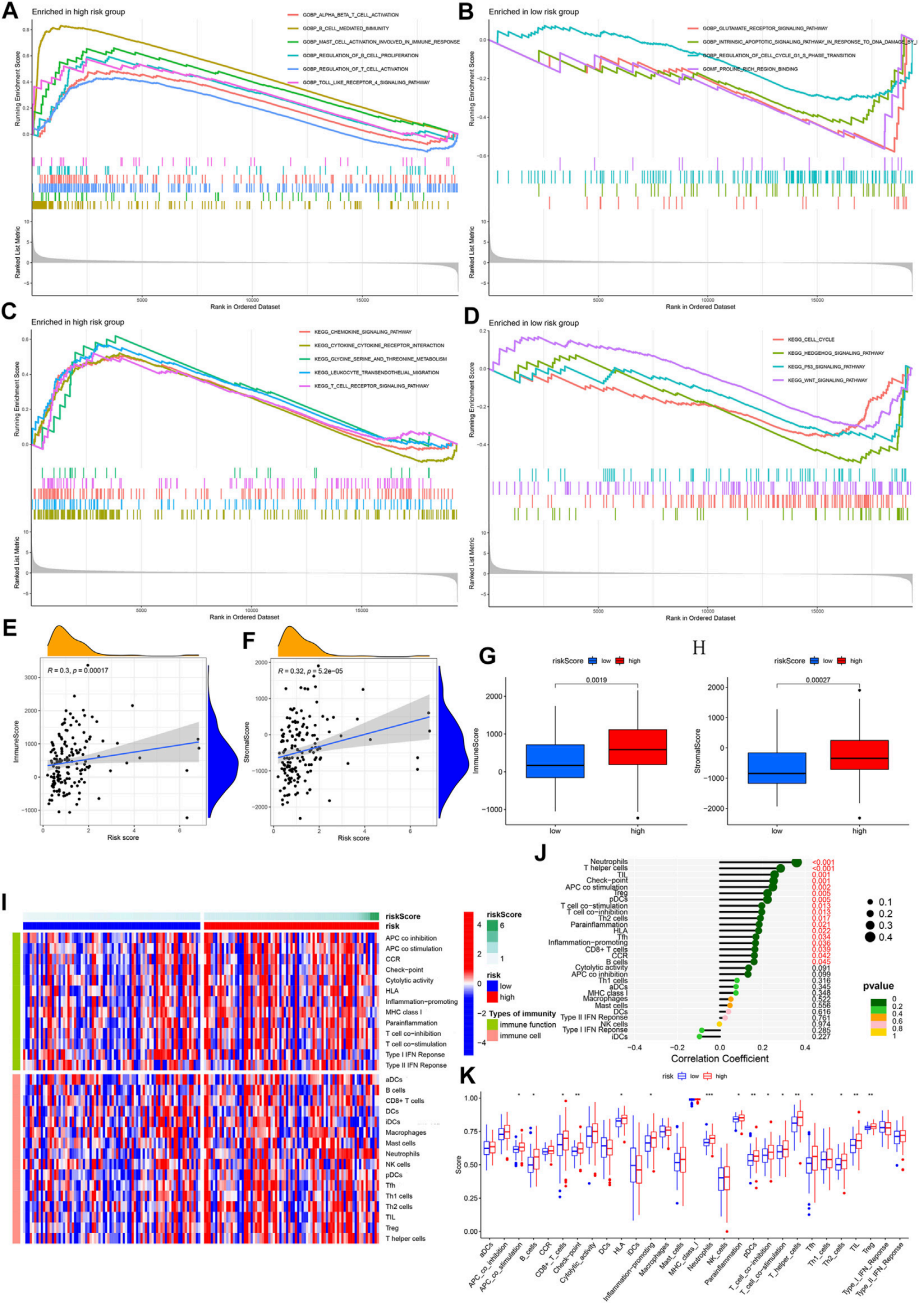
GSEA enrichment and immune infiltration assessment analyses. **(A–B)** GO enrichment analysis for high- and low-risk groups. Based on biological features. **(C–D)** GO enrichment analysis for high- and low-risk groups. Based on KEGG pathways. Color represents a pre-defined biological function or pathway. **(E–F)** Correlation analysis between risk score and immune/stromal cell score. **(G–H)** Comparison between immune/stromal cell score and high-/low-risk group. **(I)** An overview heat map of the different scores of 16 immune cells and 13 immune functions as the risk score increases. **(J)** The correlation between 16 types of immune cells, 13 types of immune function, and risk score. **(K)** Analysis of the difference between 16 types of immune cells, 13 types of immune function, and high-/low-risk groups.

Supplementary Table S2 presents the results of gene expression differences between different risk groups. The analysis results of GO-enriched cell components (CC), molecular functions (MF), and biological processes (BP) based on the high- and low-risk groups were shown in Figures 6A,B. A large number of immune-related functions were enriched in the high-risk group, including *a*-*β* T cell activation, B cell-mediated immunity, mast cell activation involved in immune response, regulation of B cell proliferation, regulation of T cell activation, and toll like receptors 4 signaling pathway. The low-risk group is mainly enriched in the glutamate receptor signaling pathway, the intrinsic apoptotic signaling pathway in response to DNA damage caused by p53 mediators, the regulation of the cell cycle G1/S phase transition, and the combination of proline-rich regions. Figures 6C,D show all the results of the KEGG pathway in the high- and low-risk groups, including chemokine signaling pathway, cytokine receptor interaction, metabolism of glycine, serine, and threonine, leukocyte transendothelial migration, and T cell receptor signaling. The pathways closely related to immunity and ferroptosis were enriched in the high-risk group.

In the process of establishing a predictive model, we have determined 121 DE-IRGs. Given that our model associated immunity, we further analyzed the immune-related risk score and the state of immune infiltration in the tumor microenvironment to determine whether the predictive model can reflect the state of the immune microenvironment. By analyzing the correlation between immune/stromal cells and risk scores, we found that immune cell scores and stromal cell scores were significantly positively correlated with risk scores (Figures 6E,F). Box plots show that the high-risk group’s immune cells score and stromal cells score are higher in value (Figures 6G,H). The heat map shows the 16 immune cell scores and 13 immune function scores status of all samples with different risk scores (Figure 6I). The correlation analysis bubble plot shows that most immune cells and immune functions positively correlated with the risk score (Figure 6J). When comparing the differences between immune cells and immune functions in high- and low-risk groups, we found that Neutrophils, T helper cells, tumor infiltrating lymphocyte (TIL), Check-point, antigen presenting cell (APC) co-stimulation, B cell, Regulatory cells (Treg), plasmacytoid dendritic cells (pDCs), T cell co-stimulation, T cell co-inhibition, Type 2 helper T (Th2) cells, human leukocyte antigen (HLA), Follicular helper T cell (Tfh), Inflammation-promoting, CD8^+^ T cells in the high-risk group are higher in value (Figure 6K). This result is consistent with the results of our correlation analysis. In summary, we have observed a link between the immune-related risk score and the tumor microenvironment.

The proportions of different immune-infiltrating cells varied from sample to sample. However, the highest proportion of T cells and macrophages could still be found (Supplementary Figure S9A). From the correlation plot, it was observed that T cell CD8 had the strongest positive correlation with T follicular helper cells and a negative correlation with macrophage M0 (Supplementary Figure S9B). The resting mast cells and neutrophils are most closely related to the activated dendritic cells (Supplementary Figure S9B).

### Prediction and Verification of ceRNA Regulatory Network

**FIGURE 7:**
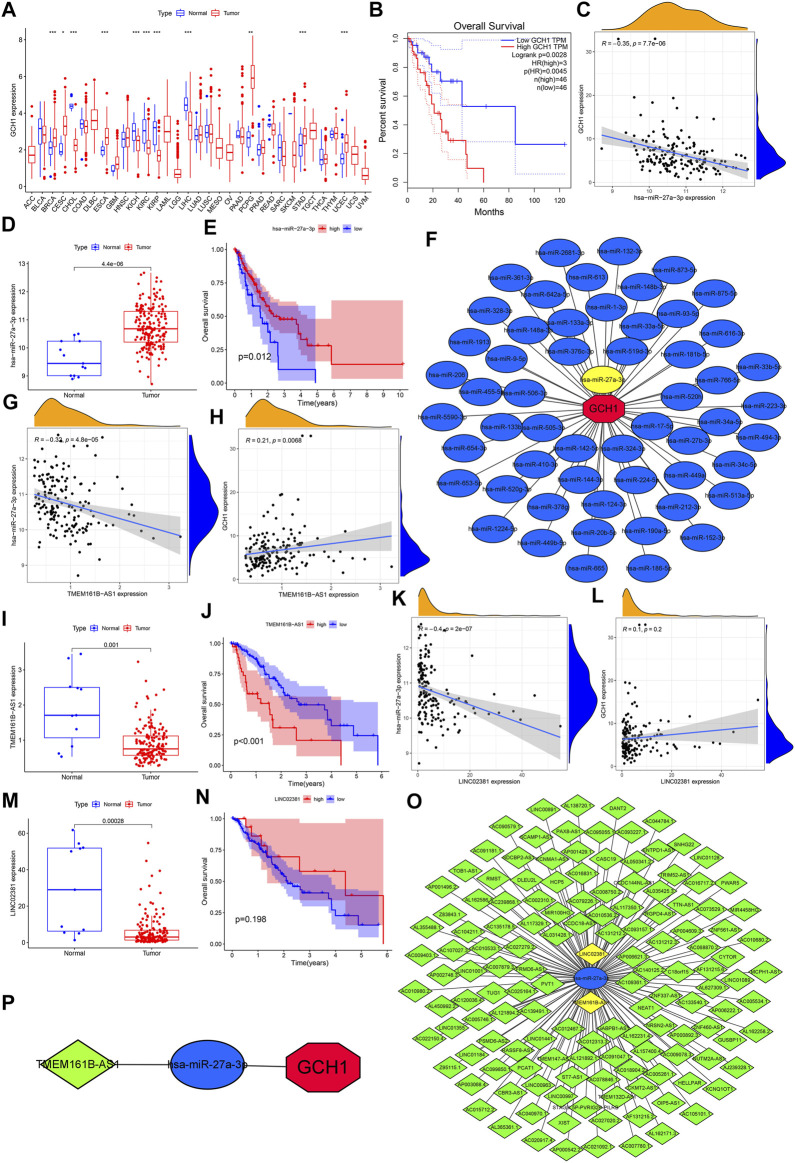
Prediction and validation of ceRNA regulatory network. **(A)** Difference in GCH1 expression between normal and tumor tissue across 33 cancer types. **(B)** Survival rates for levels of high and low GCH1 expression. **(C)** Correlation between GCH1 and hsa-miR-27a-3p expression. **(D)** Expression level of has-miR-27a-3p for normal versus ESCA tissue. **(E)** Survival rates for levels of high and low hsa-miR-27a-3p expression. **(F)** Regulatory network of miRNAs-GCH1. **(G)** Correlation between expression of TMEM161B-AS1 and hsa-miR-27a-3p. **(H)** Correlation between expression of TMEM161B-AS1 and GCH1. **(I)** TMEM161B-AS1 expression between normal and ESCA tissue. **(J)** Survival rates between groups with high and low expression of TMEM161B-AS1. **(K)** Correlation between expression of LINCO2381 and hsa-miR-27a-3p. **(L)** Correlation between expression of LINCO2381 and GCH1. **(M)** Expression levels of LINCO2381 for normal and ESCA tissue. **(N)** Survival rates between groups with high and low expression of LINCO2381. **(O)** Regulatory network of lncRNAs- hsa-miR-27a-3p. **(P)** ceRNA regulatory network genes include TMEM161B-AS1, hsa-miR-27a-3p and GCH1. The expression level of all genes refers to the transcription level of RNA.

GCH1 expression in breast invasive carcinoma (BRCA), cervica squamous cell carcinoma and endocervical adenocarcinoma (CESC), cholangiocarcinoma (CHOL), ESCA, and kidney chromophobe (KICH), kidney renal clear cell carcinoma(KIRC), kidney renal papillary cell carcinoma (KIRP), liver hepatocellular carcinoma (LIHC), pheochromocytoma and paraganglioma (PCPG), stomach adenocarcinoma (STAD), and uterine corpus endometrial carcinoma (UCEC) showed significant differences. In addition to CHOL, KICH, KIRC, KIRP, and LIHC, GCH1 expression is up-regulated in other cancers (Figure 7A). Higher GCH1 expression was found to be associated with a poorer prognosis in the GEPIA plotted survival curve (Figure 7B). We predicted the 56 upstream miRNAs that may bind to GCH1. The miRNAs-GCH1 regulatory network established by the Cytoscape software was shown in Figure 7F. According to the mechanism by which miRNA regulates the expression of target genes, there was a significant negative correlation between miRNA-27a-3p and GCH1 expression (Figure 7C). We also found that this miRNA was significantly up-regulated in ESCA (Figure 7D) with a better prognosis (Figure 7E). These findings suggest that miRNA-27a-3p may be the most promising regulatory miRNA of GCH1 in ESCA. Through the starBase database, 139 lncRNAs were obtained. The lncRNAs-GCH1 regulatory network is shown in Figure 7O. The expression of the two lncRNAs (TMEM161B-AS1 and LINC02381) were negatively correlated with the expression of miRNA-27a-3p (Figures 7G,K) and positively correlated with the expression of GCH1 (Figures 7H,L). LINC02381 and TMEM161B-AS1 were significantly less expressed in ESCA tumor tissues (Figures 7I,M). High expression of TMEM161B-AS1 indicated a worse prognosis (Figure 7J), but there was no significant difference in OS between high and low expression of LINC02381 (Figure 7N). Figure 7P shows the ceRNA regulatory network composed of TMEM161B-AS1, hsa-miR-27a-3p and GCH1.

### Analysis of the Correlation Between PR-DE-IRFeGs and Mutation Field

**FIGURE 8:**
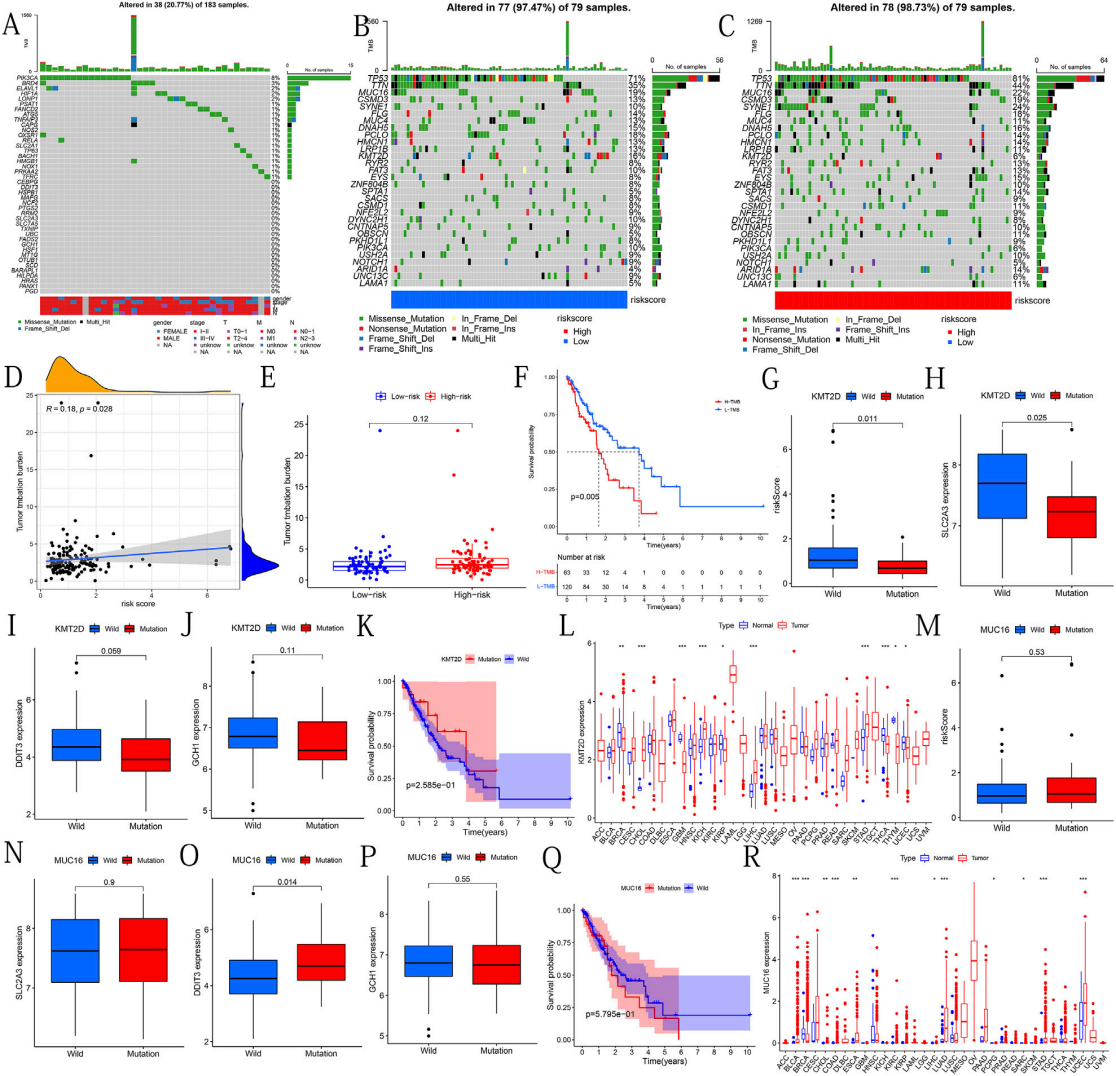
Mutational analysis of genes of interest. **(A)** Mutational types and distribution of 43 DE-IRFeGs. **(B–C)** Waterfall-type plots showing the mutation distribution of the thirty most common genes in low and high risk groups. The right panel of each plot shows the mutation frequency by type, and the color key in the bottom panel shows references to mutation type and risk score. The histogram in the top panel shows the TMB statistic for each sample. **(D)** Correlation between TMB and risk score. **(E)** TMB frequency for high- and low-risk groups. **(F)** The difference in survival rates between the high and low TMB groups. **(G)** Risk score between KMT2D wild and mutant groups. **(H–J)** Expression levels of SLC2A3, DDIT3, and GCH1 for the KMT2D wild and mutant groups. **(K)** Survival rates between KMT2D wild and mutant groups. **(L)** Expression levels of KMT2D between normal and tumor tissues across 33 cancer types. **(M)** Risk scores for MUC16 wild and mutant groups. **(N–P)** Expression levels of SLC2A3, DDIT3, and GCH1 for the MUC16 wild and mutant groups. **(Q)** Survival rates between MUC16 wild and mutant groups. **(R)** Expression levels of MUC16 for normal and tumor tissues across 33 cancers types.

We show the mutations of the 43 DE-IRFeGs through a waterfall plot (Figure 8A). Figures 8B,C show the mutations of the top 30 most common genes in 77 samples in the low-risk group and 78 samples in the high-risk group. The results showed that TMB was positively correlated with risk score (Figure 8D). However, significant differences in TMB between the high and low risk groups were not found (Figure 8E). By a Kaplan-Meier survival curve, we found that the OS of the samples with high TMB was lower (Figure 8F). Afterwards, we passed the risk score difference between the wild group and the mutant group of the KMT2D, and found that the risk score in the mutant group was lower (Figure 8G). By comparing the expression levels of the 3 PR-DE-IRFeGs genes in the KMT2D wild group and the mutant group, it was found that the expression levels of the 3 PR-DE-IRFeGs in the mutant group were all lower (only SLC2A3 shown significance, Figures 8H–J). The Kaplan-Meier survival curve shows that there is little difference in survival status between the KMT2D mutant group and the wild group (Figure 8K).

Through pan-cancer analysis, we discovered the differential expression of KMT2D between many cancers and normal tissues. KMT2D gene is highly expressed in CHOL, KICH, LIHC and STAD (Figure 8L). We used the same method to analyze the mutant gene MUC16. The results showed that there was no significant difference in the risk score and OS between the wild group and the mutant group of MUC16 (Figures 8M,Q). Except for the significantly higher expression of DDIT3 in the MUC16 mutant group (Figure 8O), no significant differences of remaining 2PR-DE-IRFeGs was found (Figures 8N,P). Pan-carcinoma results show that except in BRCA, the MUC16 is more highly expressed in other cancers tissues (Figure 8R).

### CNV Analysis

**FIGURE 9:**
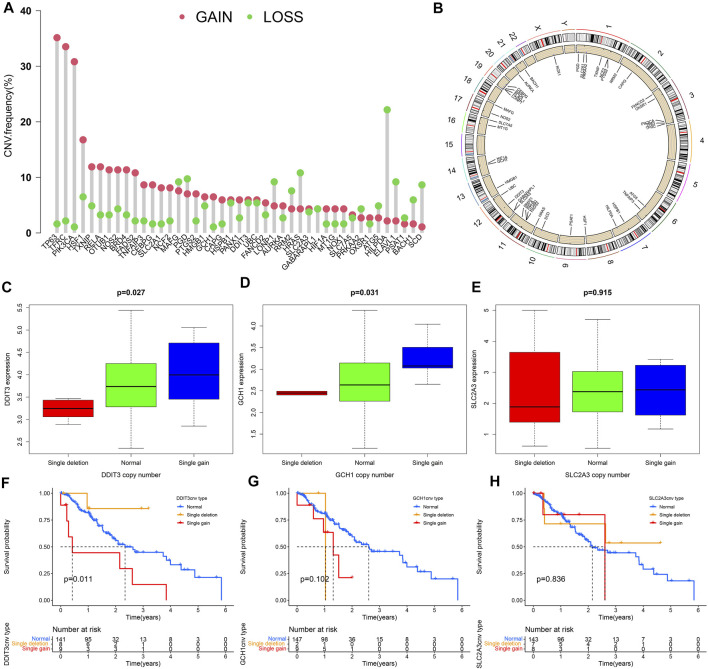
Copy number variation (CNV) analyses. **(A)** Frequency of CNV for 43 of the DE-IRFeGs. The green circle refers to copy number loss and the pink circle refers to copy number gain. **(B)** Distribution of 43 DE-IRFeG CNVs by position along the human chromosomes. **(C–E)** Three PR-DE-IRFeGs (DDIT3, GCH1, and SLC2A3) expression levels for normal, single deletion, and single gain copy number groups. **(F–H)** Survival rates among single deletion, normal, and single gain copy number groups of the three PR-DE-IRFeGs (DDIT3, GCH1, and SLC2A3), respectively.

Except for MAFG, PGD, LONP1, RRM2, HRAS, GABARAPL1, PRKAA2, ATG5, HILPDA, ELAVL1, PSAT1, BACH1, and SCD, which have a higher frequency of CNV loss, the remaining 30 DE-IRFeGs (including 3 PR-DE-IRFeGs) have a higher frequency of CNV gain (Figure 9A). Figure 9B shows the corresponding positions of these 43 genes on the chromosome and the comprehensive status of CNV. It can be observed that the CNV frequency of DE-IRFeGs on chromosomes 1, 3, 11, and 19, and the gain copy number frequency of DE-IRFeGs on chromosomes 3 and 11, is higher (Figure 9B). By comparison, the highest expression levels of DDIT3 and GCH1 were observed in the single gain copy numbers group, compared to the lowest in the single deletion copy numbers group (Figures 9C,D). Unfortunately, there was no significant difference in the expression of SLC2A3 between the three groups (Figure 9E). From the survival curve, we also observed that the survival of the single deletion copy number group of DDIT3 is the best, compared to the worst in the single gain copy number group (Figure 9F). Similar significant differences are not observed in the survival plot of GCH1 and SLC2A3 (Figures 9G,H).

### Correlation Analysis Between Predictive Model and ICIs-Related, m6A-Related and Multidrug Resistance-Related Genes

**FIGURE 10:**
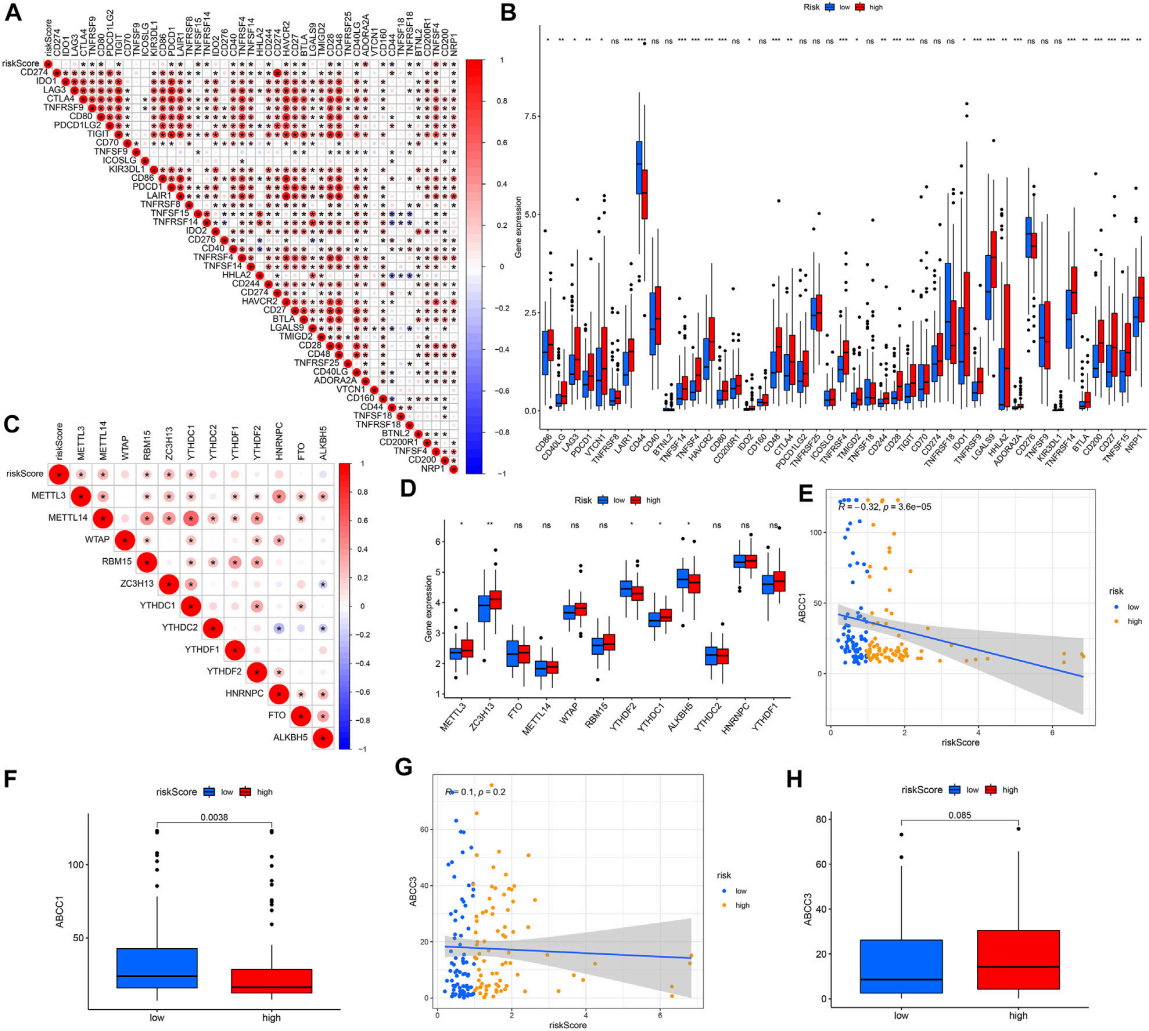
Analyses of gene correlations. **(A)** Correlation analysis between 45 ICIs-related gene expression and risk score. **(B)** Expression levels of ICIs-related genes for high- and low-risk groups. **(C)** Correlation between m6A-related genes and risk score. **(D)** Expression of m6A-related genes for high- and low-risk groups. **(E)** Correlation between ABCC1 gene expression and risk score. **(F)** Expression levels of ABCC1 for high- and low-risk groups. **(G)** Correlation between ABCC3 expression and risk score. **(H)** Gene expression of ABCC3 in high- and low-risk groups. ns, not significant; *p < 0.05; **p < 0.01; ***p < 0.001.

We analyzed the correlation between 45 ICIs-related genes and the predictive model (Figure 10A). It can be seen that CD44 and TNFRSF18 are significantly negatively correlated with the risk score, and genes such as CTLA4, TNFRSF9, CD80, TIGIT, PDCD1, etc. are positively correlated. And the difference in the expression levels of these genes between the high and low-risk groups supports our previous analysis (Figure 10B). Considering the vital role of N6-methyladenosine (m6A) in regulating mRNA splicing, export, localization, translation, and stability, we also analyzed the relationship between m6A-related genes and risk scores. The results show that METTL3, METL14, RBM15, ZC3H13, YTHDC1, and the risk score have a significant positive correlation (Figure 10C). Except for YTHDF2 and ALKBH5, METTL3, ZC3H13, and YTHDC1 which had higher expression levels in the high-risk group (Figure 10D). Finally, we also explored the correlation between the expression of drug resistance genes MRP1 (ABCC1) and MRP3 (ABCC3) and risk score. ABCC1 is negatively correlated with risk score (Figure 10E), but ABCC3 has no significant correlation with risk score (Figure 10G). The difference in expression of ABCC1 and ABCC3 between the high and low-risk groups supports our correlation analysis (Figures 10F,H).

### Clinical Application of 3 PR-DE-IRFeGs From the Predictive Model

**FIGURE 11:**
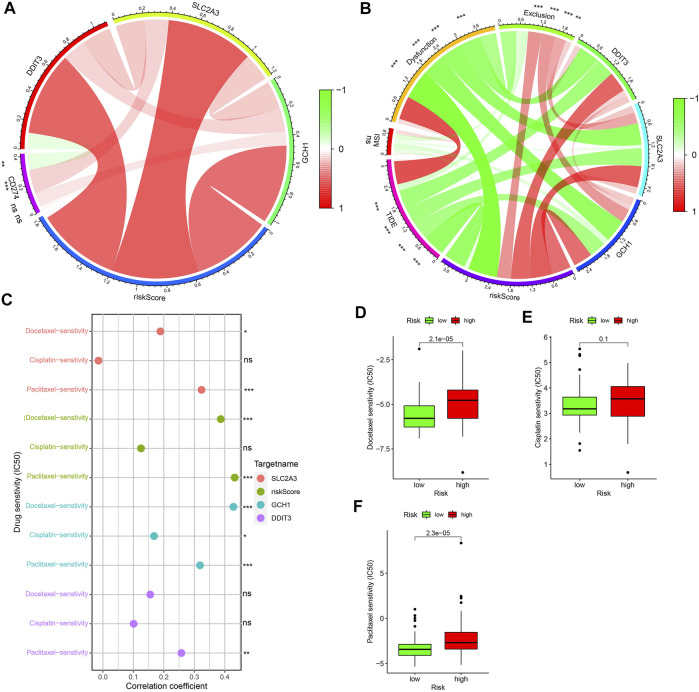
Application of model to clinical treatments. **(A)** Correlations among gene expression levels of three PR-DE-IRFeGs, CD274, and their risk score. **(B)** Correlations among gene expresion levels of three PR-DE-IRFeGs, scores for TIDE, MSI, Dysfunction, Exclusion, and risk scores. **(C)** Correlation between the three drugs in clinical use and expression level of three PR-DE-IRFeGs, risk score. **(D–F)** Drug sensitivity of docetaxel, cisplatin, and paclitaxel for the high and low-risk groups.

The circle graph shows that SLC2A3 is positively correlated with CD274, while DDIT3 is negatively correlated with CD274 (Figure 11A). Higher tide prediction score represents a higher possibility of immune escape, which indicates that the patient is less likely to benefit from ICIs treatment (Jiang et al., 2018). Figure 11B shows the correlation between TIDE, MSI, Dysfunction, Exclusion, and risk score/the three PR-DE-IRFeGs. 3 PR-DE-IRFeGs/risk score show a significant negative correlation with TIDE and dysfunction, and a significant positive correlation with Exclusion, indicating that our model containing 3 PR-DE-IRFeG has greater application value in immunotherapy. Docetaxel and paclitaxel were observed positively correlated with SLC2A3 and risk score (Figure 11C). A positive correlation between GCH1 and these three drugs, and a positive correlation between DDIT3 and paclitaxel were also observed (Figure 11C). The difference in IC50 of the three drugs between the high and low-risk groups also supports our correlation analysis (Figures 11D–F).

### Construction and Verification of Forecast Nomogram

Supplementary Figure S10A shows the nomogram constructed by 2 clinical prognostic factors (N staging and M staging) and risk score. The time-dependent ROC curve shows the nomogram’s excellent predictive performance in 1, 2, and 3- years of OS (majority of AUC > 0.65, Supplementary Figures S10B–D). We have observed similarities between the predicted OS and the actual OS in most years based on 3 data sets (Supplementary Figures S10E–G). These data indicate that the nomogram has a better ability to predict OS in patients with ESCA.

## Discussion

As a common and highly heterogeneous malignant tumor (Fisher et al., 2013), ESCA lacks accurate biomarkers to predict the survival prognosis of patients. Four ESCA datasets from TCGA and GEO databases were used to screen out three PR-DE-IRFeGs for constructing a predictive model. After multiple analysis and verification of multiple internal and external datasets, our model has proven to meet this requirement. In addition, our model showed the best predictive value compared with other models in previous research (Song et al., 2021; Tang et al., 2021). Three PR-DE-IRFeGs have been reported to be closely related to ferroptosis and immunity, as well as the occurrence, development and prognosis of certain cancers. Through in-depth exploration from multiple perspectives, many potential roles of the immune system and ferroptosis in ESCA have been observed. At present, immunotherapy has attracted much attention in the treatment of patients with advanced ESCA, and the optimal treatment plan is particularly important in the multimodal treatment of ESCA. The close correlation between clinical treatment sensitivity and the model also demonstrates the excellent guiding value of our model in immunotherapy and chemotherapy. Not only that, the high correlation between the model and genes related to multidrug resistance, M6A, and ICIs also implies a close relationship among them. Finally, a nomogram composed of comprehensive factors was constructed to accurately and efficiently predict the survival rate of cancer patients.

The three PR-DE-IRFeGs (DDIT3, SLC2A3, and GCH1) used to construct the model were all identified as risk factors. Solute carrier family 2(facilitated glucose transporter), member 3 (SLC2A3) encodes glucose transporter 3 (GLUT3), which can inhibit ferroptosis and is closely related to the poor prognosis of cancer (Masin et al., 2014). Dai et al. (2013) found that highly expressed miR-106a can hinder the effect of SLC2A3 and further inhibit cell proliferation and glycolysis in gliomas. In addition, the up-regulation of the SLC2A3 gene has also been observed to reduce the OS and Disease Free Survival of patients with colorectal cancer (Gao et al., 2021). As a transcription factor that induces DNA damage, recombinant DNA damage inducible transcript 3 (DDIT3) can develop diseases through apoptosis and autophagy (Liu H. et al., 2021). Tan et al. (2016) found that DDIT3 was significantly up-regulated in T-47D breast cancer cells, which promoted the formation of endoplasmic reticulum and autophagosomes, and ferritin autophagy mediated by NCOA4 could control cellular iron homeostasis to support ferroptosis (Yang M. et al., 2019). These results indicated that DDIT3 could affect ferroptosis by affecting autophagy. GTP cyclized hydrolase 1 (GCH1) is the rate-limiting enzyme in the biosynthesis of tetrahydrobiopterin (BH4) (Zhang et al., 2007). Wei et al. (2020) found that cells with high expression of GCH1 induce lipid remodeling by synthesizing BH4/BH2, forming a GCH1-BH4-phospholipid axis to inhibit ferroptosis is related to the poor prognosis of glioma patients. These results all supported that the three PR-DE-IRFEGs are closely related to ferroptosis and immunity. Similar to these studies, poor prognoses were observed in the up-regulation of these genes in ESCA, which may also be caused by the suppression of iron death.

To further understand the biological functions and molecular mechanisms of 43 DE-IRFeGs, GO function and KEGG pathway enrichment analysis were performed. The results showed that the BPs, MFs, and CCs enriched in DE-IRFeGs were mainly related to hypoxia and cell autophagy, such as oxidative stress and the response to oxygen levels from GO enrichment. Similarly, pathways related to autophagy, such as autophagy-animal, mitophagy-animal, HIF-1 signaling pathway, etc, have also been discovered. Li et al. (2019) found that the form of carbonic anhydrase 9 inhibiting multiple myeloma cell death under hypoxic conditions is mainly a mixed cell death of apoptosis and ferroptosis through autophagy process. In addition, studies have also found that cytoplasm actively controls ferroptosis by interacting with GPX4 to activate the autophagy degradation of GPX4 (Chen C.a. et al., 2021). After combining these studies, we considered that the screened DE-IRFeGs were closely related to ferroptosis and autophagy.

To verify the utility of our model, we compared our model, the ferroptosis-related model of Song et al. (2021) and the autophagy-related model of Tang et al. (2021). After comparing the ROC plot and Kaplan-Meier survival plot, it is found that our model has the best performance in predicting the prognosis of ESCA patients. Compared with the other two studies, our study also shows other advantages. Compared with the model constructed by Song et al. (2021) which is based on two data sets, we used four data sets to screen for differential genes and used co-expression analysis to identify DE-IRFeGs as incorporated in our model. As compared with the lack of analysis of prognostic factors, such as immune infiltration in the predictive model constructed by Tang et al. (2021), we have established some interesting findings. And by the c-index comparison, we observe that our model performs better than Tang et al.’s model and has the highest RMST curve among the three models of interest. These analyses support the utility of our predictive model. Considering the joint role of immunity and ferroptosis in tumor development, and our analysis of their genetics (IRGs and FRGs), we consider our approach more favorable to studies which examine only one of these roles (see Guo et al., 2020; Zhang et al., 2021). Our model also has predictive value for chemotherapy and immunotherapy, and shows good performance across multiple internal and external datasets, both features not included in Guo et al. (2020) and Zhang et al. (2021).

The immune microenvironment of cancer cells plays an essential role in inhibiting tumor proliferation or promoting tumor progression (Tang et al., 2021). We observed a large number of biological pathways and processes related to immune cells enriched in the high-risk group, which suggested that there may be immune microenvironment-related biological processes in the high-risk group. The positive correlation between immune and stromal cell scores and risk scores observed in further immunological analysis supports this conclusion. We also observed higher neutrophils, T helper cells, B cells, Tregs, pDCs, T cell costimulation, HLA, Tfh, inflammation promotion, and CD8^+^ T cell scores in the high-risk group. Many studies have shown that the increase of neutrophils in tumor tissues is related to the poor prognosis of patients. For example, Hanne Krogh Jensen et al. Jensen et al. (2009) found that neutrophils in tumors are poor prognostic factors for renal cancer. Niels Borregaard’s research results also shown that high neutrophils are associated with poor overall survival (Borregaard, 2010). Inflammation and the development of ESCA seem to be closely related (Abdel-Latif et al., 2009). In the high-risk group, Neutrophils, Treg, Inflammation-promoting CD8^+^ T cells are higher than those in the low-risk group. These cells also dominate the inflammation response (Zhao et al., 2016), which explains why their content is higher in the high-risk group. Wang et al. (2019) found that CD8^+^ T cells release cytokines, including tumor necrosis factor and interferon *γ*, to drive tumor cell killing by regulating ferroptosis. Co-stimulation has been found to promote the proliferation and survival of CD8^+^ T cells and Tregs (Chen and Flies, 2013). Combining our results, we suspect that increased T cell costimulation in patients in the high-risk group may promote the proliferation of CD8^+^ T cells and Tregs, increasing their content in ESCA. The increased CD8^+^ T cells release interferon γ to improve the response of interferon γ to regulate ferroptosis. These results support the potential role of immunity and iron death in the progression of ESCA.

In recent years, as the understanding of RNA function has gradually deepened, more and more studies have confirmed that miRNA, lncRNA, and other RNAs play an essential role in regulating tumors and immunity (Tay et al., 2014). Although many studies have explored the impact of immune-related genes on ESCA, the role of immune-related miRNA and lncRNA in the progression of ESCA has not been clearly explained, especially the analysis based on high-throughput sequencing has been lacking. Therefore, it is significant to explore the potential regulatory mechanism of the ceRNA network composed of mRNA-miRNA-lncRNA in the progress of ESCA. After prediction and verification of RNA sequencing data, the ceRNA regulatory network composed of TMEM161B-AS1, hsa-miR-27a-3p and GCH1 was screened out, which may play an important biological role in ESCA.

Through the differential analysis of expression in pan-cancer, GCH1 was observed to be up-regulated in most tumor tissues. Recently, many studies have shown that the high expression of GCH1 associated with the poor prognosis of tumors. Wei et al. (2021) found that the expression of GCH1 was positively correlated with the penetration of Tregs, and high GCH1 expression was related to the decrease in the overall survival rate of triple negative breast cancer. (Tran et al., 2018) also confirmed that higher levels of GCH1 in patients with gliomas are related to higher grades of gliomas, recurrence and poor survival rates. The study by Gitanjali Pickett found that inhibiting or silencing GCH1 will reduce the proliferation and survival of tumor cells, and the expression of GCH1 will increase under hypoxia (Pickert et al., 2013). These results are consistent with the better prognosis we have observed in the low expression of GCH1 in ESCA. Data from TCGA also confirmed that the expression of upstream miR-27a-3p was significantly negatively correlated with GCH1, but positively correlated with prognosis. A study by Zhu K.-P. et al. (2019) have also found that miR-27a can inhibit ESCC tumorigenesis by targeting KRAS. Yan et al. (2019) found that miR-27a-3p has the function of a tumor suppressor, regulates the proliferation of non-small cell lung cancer cells by targeting HOXB8, and plays a tumor suppressor effect in non-small lung cancer.

Similarly, data from TCGA also confirmed that the expression of upstream TMEM161B-AS1 was significantly negatively correlated with miR-27a-3p and prognosis. The results have been confirmed in other studies. Chen Q. et al. (2021) found that lncRNA TMEM161B-AS1 mediated by HSA-Mir-27a-3p had an inhibitory effect on glioma cells, and hsa-Mir-27a-3p inhibited the proliferation, migration, and invasion of glioma cells by down-regulating the expression of FANCD2 and CD44, thus promoting cell apoptosis and ferroptosis. This supports that miR-27a-3p can target TMEM161B-AS1. Based on the above results, we infer that the overexpression of TMEM161B-AS1 may up-regulate the expression of GCH1 by competitively binding hsa-miR-27a-3p to promote the proliferation, migration, and invasion of ESCA cells.

Gene mutations play an important role in the occurrence, development and prognosis of tumors. As a response to the number of mutations, TMB can be used as a marker to predict the effect of immunotherapy in cancer patients. It was observed that TMB was positively correlated with risk score and negatively correlated with prognosis in our study. Among the 30 most commonly mutated genes in ESCA, KMT2D was observed to have a higher mutation frequency. Through analysis, we found that the risk score of the KMT2D mutation group was lower. The KMT2D gene encodes histone methyltransferase to methylate the Lys-4 position of histone H3. It has been found that KMT2D is closely related to tumor cell migration and adhesion. The KMT2D mutation caused by the loss of KMT2D will inhibit tumor migration, which is beneficial to the prognosis of cancer (Guo et al., 2013). Interestingly, recent studies have reported that KMT2D mutations are inhibitors of the development of ESCA (Zhang et al., 2020). In addition, KMT2D mutations have also been observed to have a longer survival time in patients with small-cell lung cancer (Simbolo et al., 2017). Through analysis, we found a lower risk score and a better prognosis in the KMT2D mutation group in ESCA. Combining these results, we suspect that the KMT2D mutants may play a role in ESCA through and PR-DE-IRFeGs related to risk scores. The significantly lower SLC2A3 expression in the KMT2D mutant group supports this conjecture. The critical target that mediates the function of KMT2D inhibition in tumors by whole-genome analysis is also SLC2A3 (Koutsioumpa et al., 2019). According to existing studies, it is found that SLC2A3 has a high affinity for glucose, which can ensure the effective uptake of glucose by cells (Simpson et al., 2008), and the low expression of KMT2D significantly affects the effect of SLC2A3, thereby inhibiting cancer cells’ uptake and utilization of glucose. The functional characteristics shown by the reduction of SLC2A3 in KMT2D mutant group indicate that KMT2D mutation may be closely related to the down-regulation of the ferroptosis-related gene SLC2A3 and the promotion of ESCA progression.

With the emergence of large-scale sequencing, it has become a trend to study diseases from molecular mechanisms. At present, studies have shown that the correlation between gene expression and CNV has a biological effect on the occurrence and development of cancer (Heitzer et al., 2016). CNV causes the heterogeneity of cancer genes. And CNV can be used to diagnose specific tumor subtypes (Friedman et al., 2009) for early clinical diagnosis and early intervention of tumors. Since CNV may lead to genetic instability, increased genomic instability is associated with the poor prognosis of many cancer types (Shi et al., 2012; Tanenbaum et al., 2016). SLC2A3 duplication is a frequently detected CNV phenomenon. Studies have shown that SLC2A3 duplication may be a genetic modifier of individual congenital heart defects and aortic arch abnormalities. The loss of 22q11.2 CNV leads to the repetitive expression of SLC2A3, causing abnormal glucose transport, affecting the development of the heart and the production of diseases (Mlynarski et al., 2015). The expression levels of DDIT3 and GCH1 were the highest in the single gain copy group, while the survival rate in DDIT3 single deletion group was the worst in ESCA. Combined with our research results, we infer that CNV may play a role in ESCA by affecting the expression of DDIT3 and GCH1.

Given the vital role of ICIs for metastatic systemic anti-tumor therapy (Das et al., 2020), we analyzed the correlation between the predictive model and ICIs. ICIs are a class of biological agents that can promote immune cells to fight tumors by interacting with the immune system and respond to tumors by changing the immune microenvironment to change the state of immune infiltration (Derakhshani et al., 2021). The expression of many ICIs-related genes was found to be significantly correlated with our risk score. CTLA4 and PDCD1 genes are two representative immune checkpoint genes, proven to have an excellent immune blocking effect in various cancers (So et al., 2020). These immune checkpoint suppressor genes were observed higher in the high-risk group, meaning high-risk patients with ESCA in the group are more suitable for immunotherapy with corresponding ICIs. Research in recent years has shown that M6a methylation is a reversible RNA modification process. By detecting changes in m6A-regulated gene expression (Wang et al., 2020), the relationship between m6A status and the development of tumor diseases can be assessed. In addition, PD1/PD-L1 checkpoint blockade is regulated by YTHDF1 (m6A reader) and FTO (eraser), and m6A modulators may be potential anti-cancer immunotherapy targets (Yang S. et al., 2019; Han et al., 2019). METTL3 promotes the growth and tumorigenesis of acute myeloid leukemia cells and inhibits renal cell carcinoma (Zhang C. et al., 2017; Ianniello et al., 2019). ZC3H13 can inhibit the proliferation and invasion of colorectal cancer and regulate the self-renewal of mouse embryonic stem cells (Zhu D. et al., 2019). YTHDC1 can retain oncogene mRNA in the nucleus and help to eliminate abnormally mutated malignant cells subsequently. These genes are all up-regulated in the high-risk group of ESCA. Multidrug resistance is the main obstacle to the success of ESCA chemotherapy. The correlation between risk score and multidrug resistance genes also provides ideas and guidelines for clinical treatment. These results all demonstrate the guiding value of our model in multiple fields.

ICIs mainly represented by PD-L1 (CD274) have shown great value in researching and treating various malignant tumors (Nishino et al., 2017). PD-1 inhibits these immune checkpoints by binding to PD-L1 inhibitors, promotes tumor immune responses of T cells, and exhibits anti-tumor effects (Zhang M. et al., 2017). We found a significant positive correlation between CD274 and SLC2A3, indicating that ESCA patients with high-expressing SLC2A3 can benefit more from CD274 immunotherapy. TIDE is composed of genome-wide T cell dysfunction and rejection scores. Patients with higher TIDE scores have a higher chance of anti-tumor immune escape, thus showing a lower immune checkpoint blockers (ICB) treatment response rate (Jiang et al., 2018). It can be used to predict tumors before treatment characteristics predict the clinical response of ICB (Jiang et al., 2018). We found that TIDE is negatively correlated with a risk score, which means that high-risk ESCA patients with lower TIDE scores have more favorable responses to ICB. In our study, higher PD-L1 and lower TIDE had better effects in immunotherapy in the high-risk group. These results suggest that our model can be used as a marker for the efficacy of immunotherapy. In addition, the sensitivity of chemotherapy drugs is also an essential evaluation of clinical treatment. The risk score and the 3 PR-DE-IRFeGs genes are significantly correlated with the 3 chemotherapy drugs recommended by the NCCN guidelines, indicating that the expression of these genes may substantially enhance the treatment effect of these drugs for patients with ESCA.

Through rigorous screening based on multiple datasets, we filter out PR-DE-IRFeGs through co-expression analysis for constructing a novel predictive model, which fills the gap in signature of the immune-related ferroptosis gene. Our research has also unearthed the potential biological processes in ESCA from multiple levels, which may provide some meaningful starting points for follow-up research. Although the model still maintains excellent performance and clinical application value under repeated verification, there are still many shortcomings in our research. First of all, as a retrospective analysis of shared data, the model’s actual clinical value needs to be tested in practice. Limited data sources and sample size affect the accuracy of our analysis’ results. For this reason, we have worked hard to discover additional datasets and data types for our analysis. The limited data types also bring great challenges to the completeness and accuracy of the conclusions of many mechanisms in our analysis. However, we still employed complex and comprehensive analyses with limited data to provide more reliable support for our conclusions. Secondly, limited by the small number of FRGs and the need to meet sufficient PR-DE-IRFeGs for subsequent analysis, we were unable to incorporate strict fold changes to filter FRGs in our differential analysis.

## Data Availability

The datasets presented in this study can be found in online repositories. The names of the repository/repositories and accession number(s) can be found in the article/Supplementary Material.

## Glossary

GEO: Gene Expression Omnibus
TCGA: The Cancer Genome Atlas database
IRGs: immune-related genes
FRGs: ferroptosis-related genes
DE-IRGs: differentially expressed immune-related genes
DE-FRGs: differentially expressed ferroptosis-related genes
DE-IRFeGs: differentially expressed immune-related ferroptosis genes
PR-DE-IRFeGs: prognostic-related differential expressed immune-related ferroptosis genes
GO: Gene Ontology
KEGG: Kyoto Encyclopedia of Genes and Genomes
ROC: Receiver Operating Characteristic
C-index: concordance index
RMST or RMS: restricted mean survival time
ICIs: immune checkpoint inhibitors
m6A: N6-methyladenosine
TIDE: Tumor Immune Dysfunction and Exclusion
GSEA: Gene Set Enrichment Analysis
TIME: Tumor immune microenvironment
ceRNA: competing endogenous RNAs
CNV: Copy Number Variation
ESCA: esophageal cancer
ESCC: esophageal squamous cell carcinoma
miRNA: microRNA
N: normal: N staging
AUC: area under the curve
T: T staging
M: M staging
N: normal: N staging
IRFR: immune-related ferroptosis related
HR: hazard ratio
R: Correlation coefficient
APC: antigen presenting cell
CCR: Chemokine Receptor
HLA: human leukocyte antigen
DC: dendritic cell
iDC: immature dendritic cells
pDC: plasmacytoid dendritic cells
NK: natural killer cell
Tfh: Follicular helper T cell
Th1 cells: Type 1 helper T cells
Th2 cells: Type 2 helper T cells
TIL: tumor infiltrating lymphocyte
Treg: Regulatory cells
ACC: Adrenocortical carcinoma
BLCA: Bladder Urothelial Carcinoma
BRCA: Breast invasive carcinoma
CESC: Cervical squamous cell carcinoma and endocervical adenocarcinoma
CHOL: Cholangiocarcinoma
COAD: Colon adenocarcinoma
DLBC: Lymphoid Neoplasm Diffuse Large B-cell Lymphoma
GBM: Glioblastoma multiforme
HNSC: Head and Neck squamous cell carcinoma
KICH: Kidney Chromophobe
KIRC: Kidney renal clear cell carcinoma
KIRP: Kidney renal papillary cell carcinoma
LAML: Acute Myeloid Leukemia
LGG: Brain Lower Grade Glioma
LIHC: Liver hepatocellular carcinoma
LUAD: Lung adenocarcinoma
LUSC: Lung squamous cell carcinoma
MESO: Mesothelioma
OV: Ovarian serous cystadenocarcinoma
PAAD: Pancreatic adenocarcinoma
PCPG: Pheochromocytoma and Paraganglioma
PRAD: Prostate adenocarcinoma
READ: Rectum adenocarcinoma
SARC: Sarcoma
SKCM: Skin Cutaneous Melanoma
STAD: Stomach adenocarcinoma
TGCT: Testicular Germ Cell Tumors
THCA: Thyroid carcinoma
THYM: Thymoma
UCEC: Uterine Corpus Endometrial Carcinoma
UCS: Uterine Carcinosarcoma
UVM: Uveal Melanoma
MSI: Microsatellite instability: Microsatellite Instability
OS: Overall survival: Overall Survival
FC: fold change
FDR: false discovery rate
BP: Biological Process
MF: Molecular Function
CC: Cellular Component
Futime: overall survival
Fustat: survival state
P: P value
Stage: clinical stages
ssGSEA: single-sample gene set enrichment analysis
TMB: Tumor mutation burden
ICB: immune checkpoint blockers
IC50: half-maximal inhibitory concentration
GDSC: Cancer Drug Sensitivity Genomics
NNCN: National Comprehensive Cancer Network
MSI: Microsatellite instability: Microsatellite Instability
OS: Overall survival: Overall Survival
