# Supplementary figures and images for: Novel Immune-Related Ferroptosis Signature in Esophageal Cancer: An Informatics Exploration of Biological Processes Related to the TMEM161B-AS1/hsa-miR-27a-3p/GCH1 Regulatory Network
Source: Front Genet. 2022 Feb 24;13:829384. doi: 10.3389/fgene.2022.829384 (PMC8908453; doi:10.3389/fgene.2022.829384)

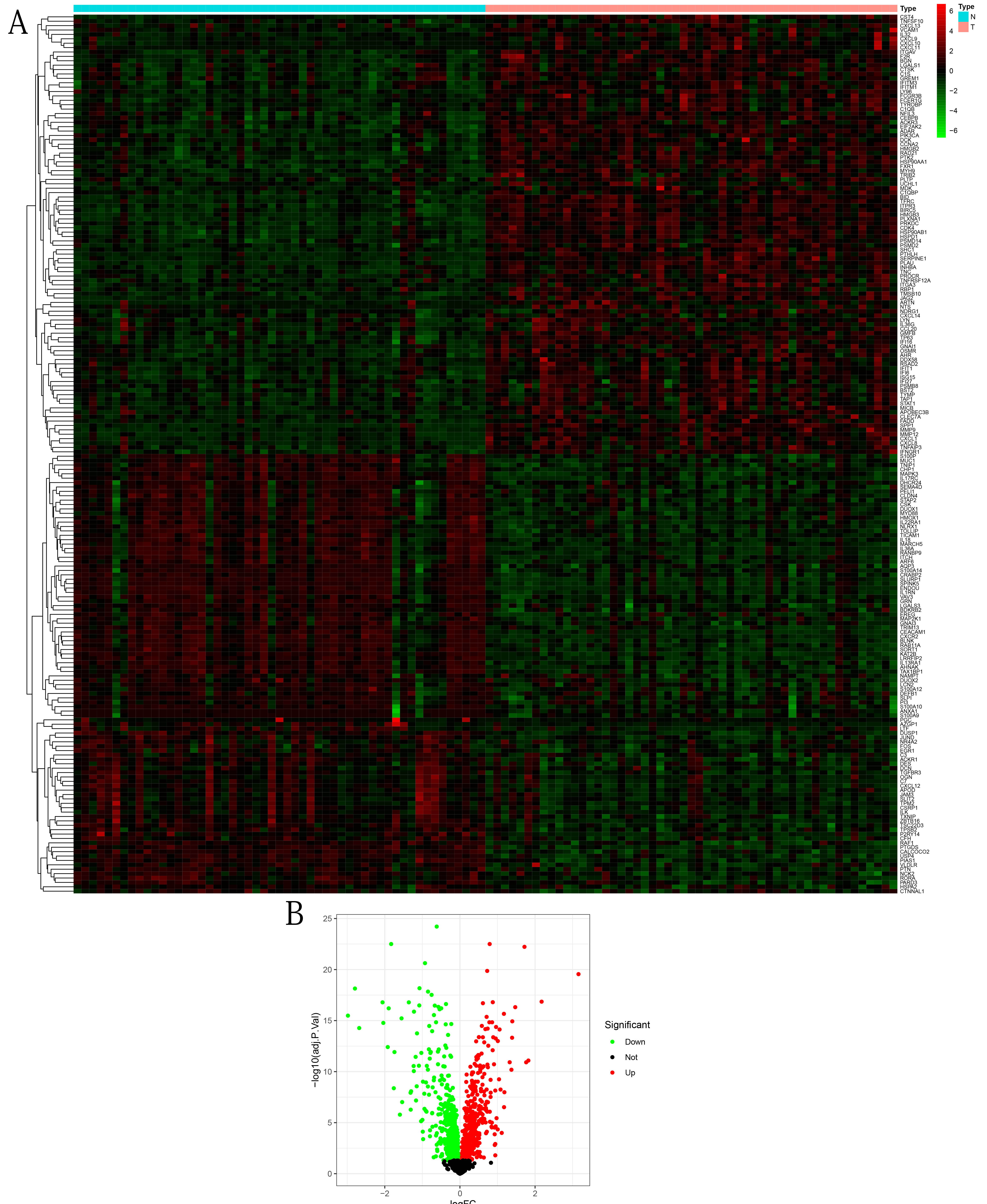

Supplement: Supplementary file 2 [file Image3.JPEG]

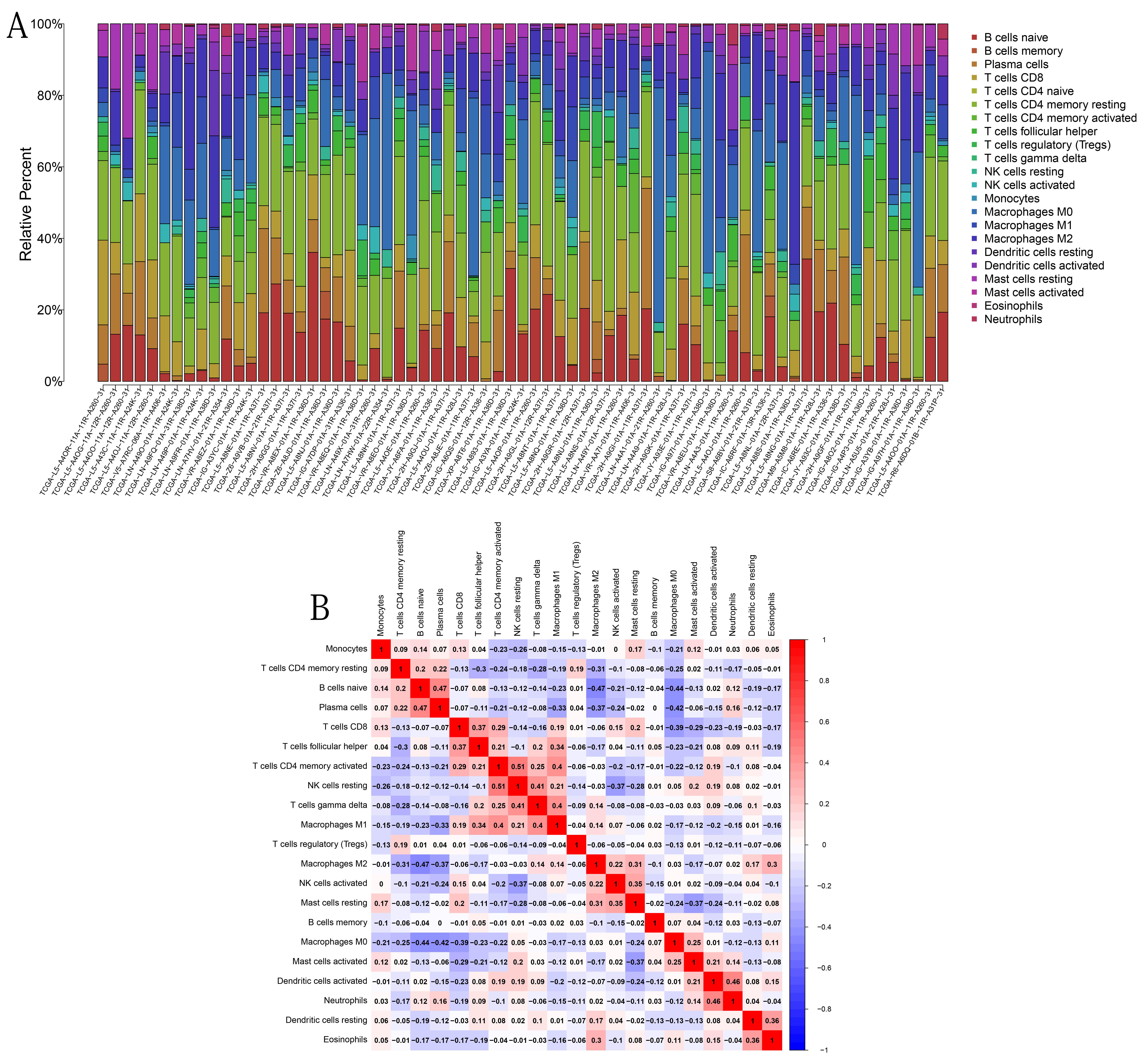

Supplement: Supplementary file 4 [file Image9.JPEG]

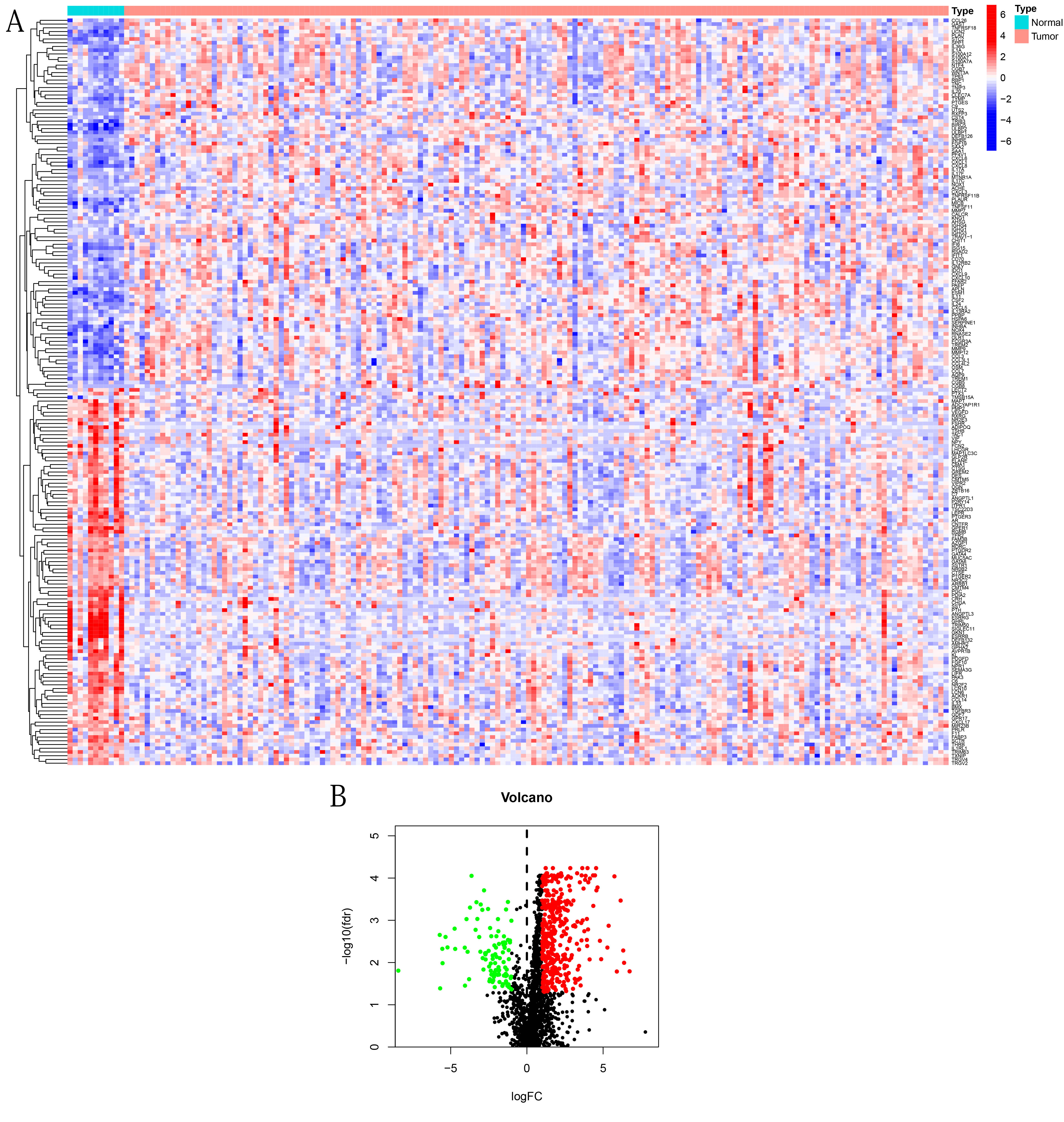

Supplement: Supplementary file 5 [file Image1.JPEG]

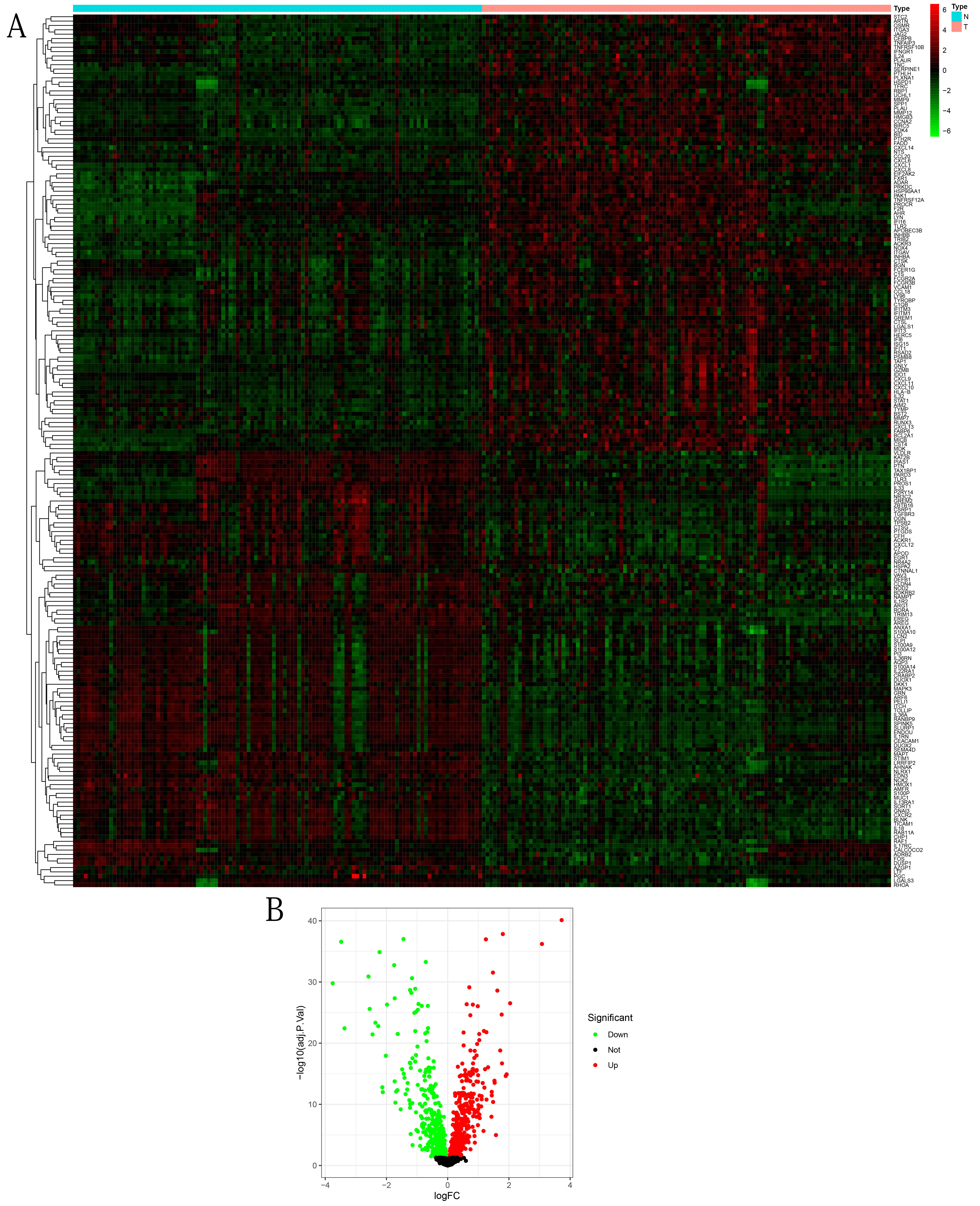

Supplement: Supplementary file 6 [file Image4.JPEG]

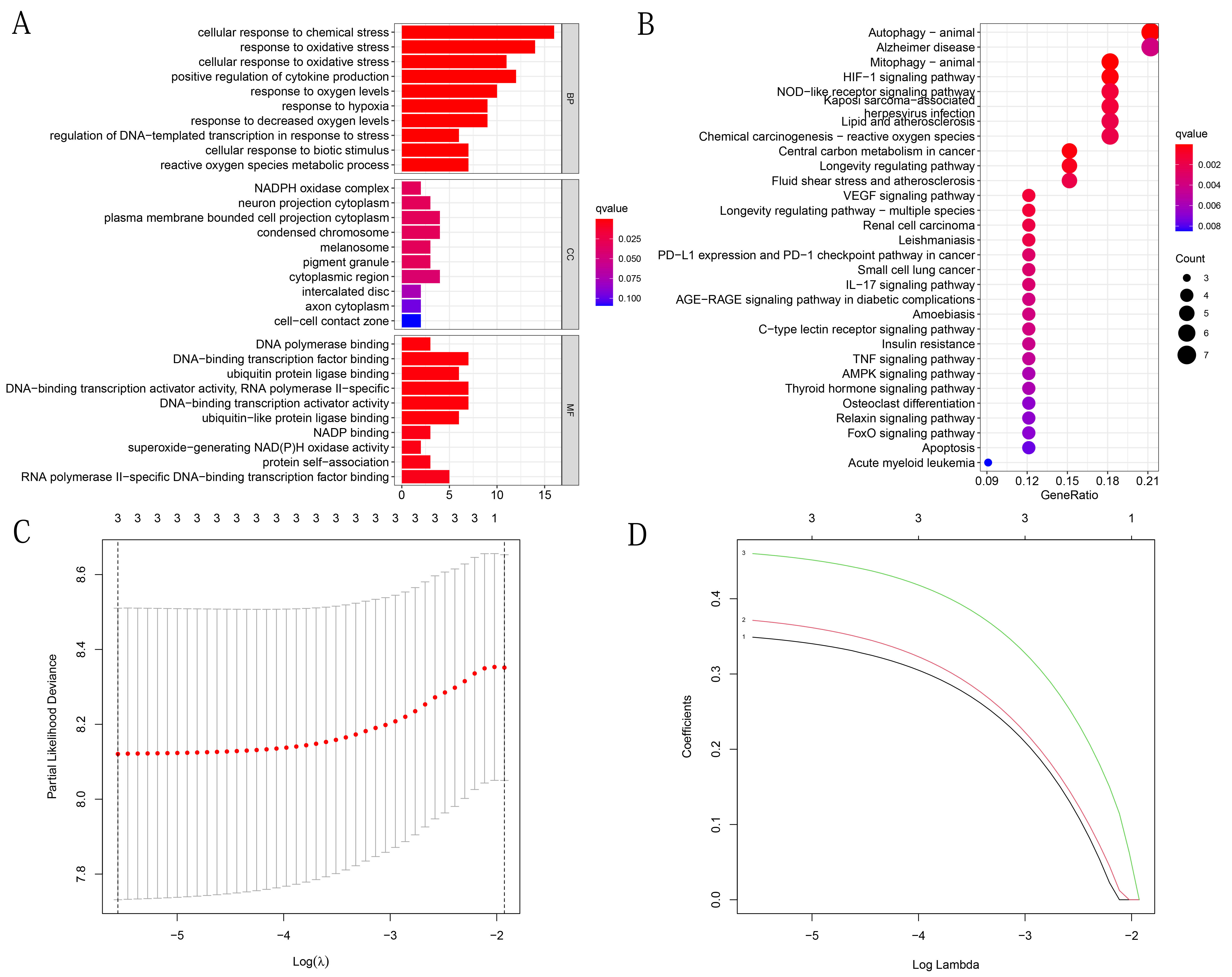

Supplement: Supplementary file 7 [file Image7.JPEG]

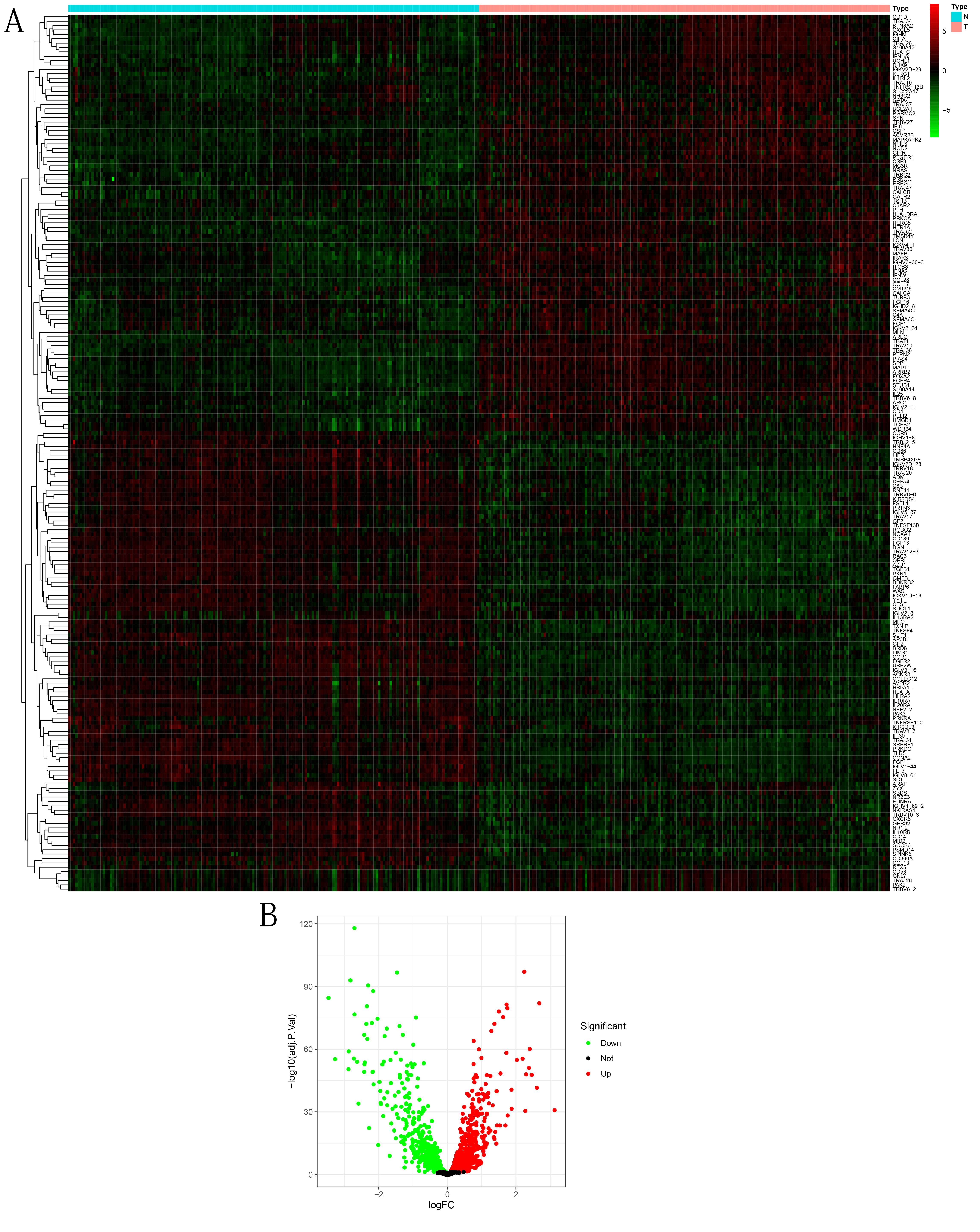

Supplement: Supplementary file 8 [file Image2.JPEG]

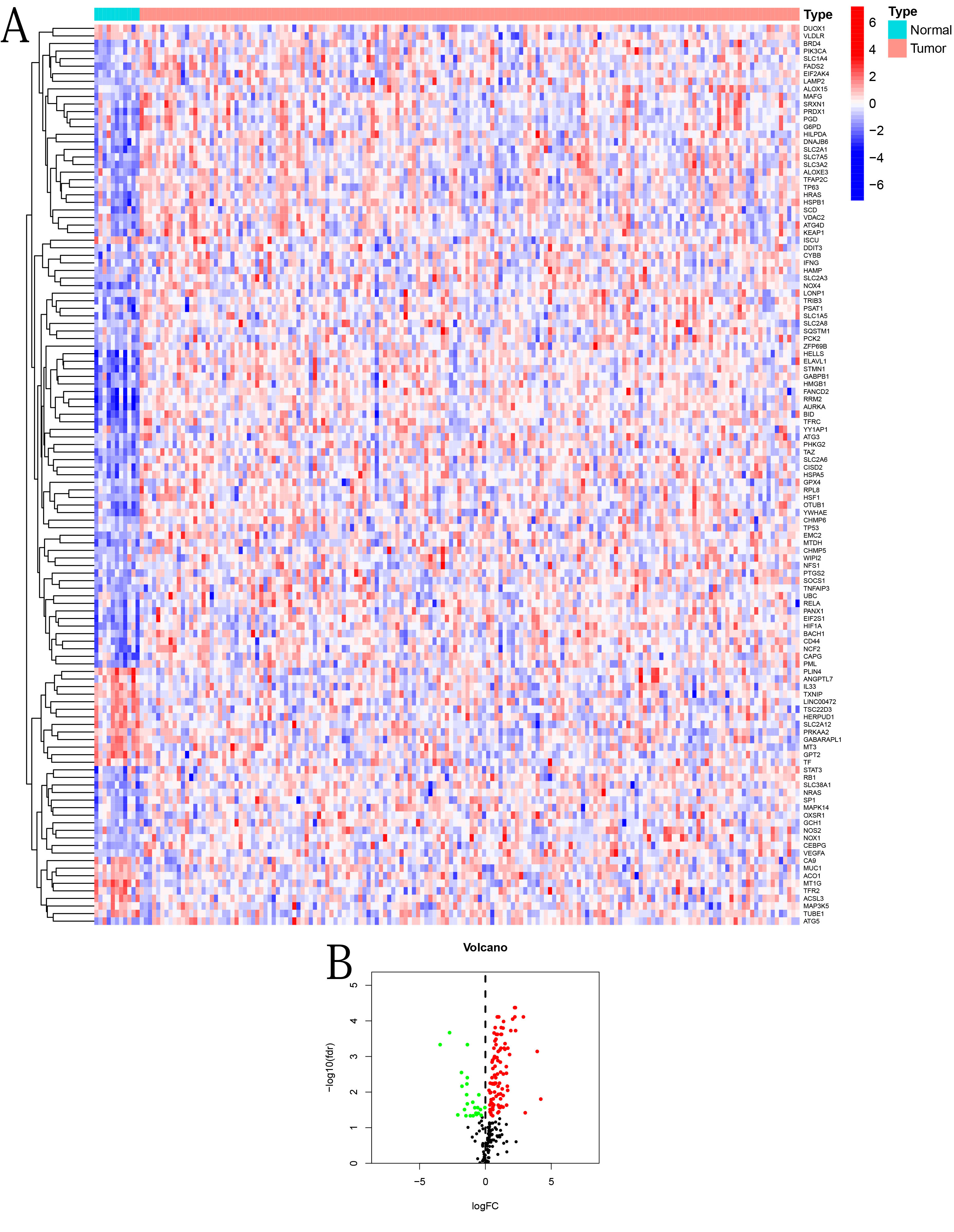

Supplement: Supplementary file 9 [file Image5.JPEG]

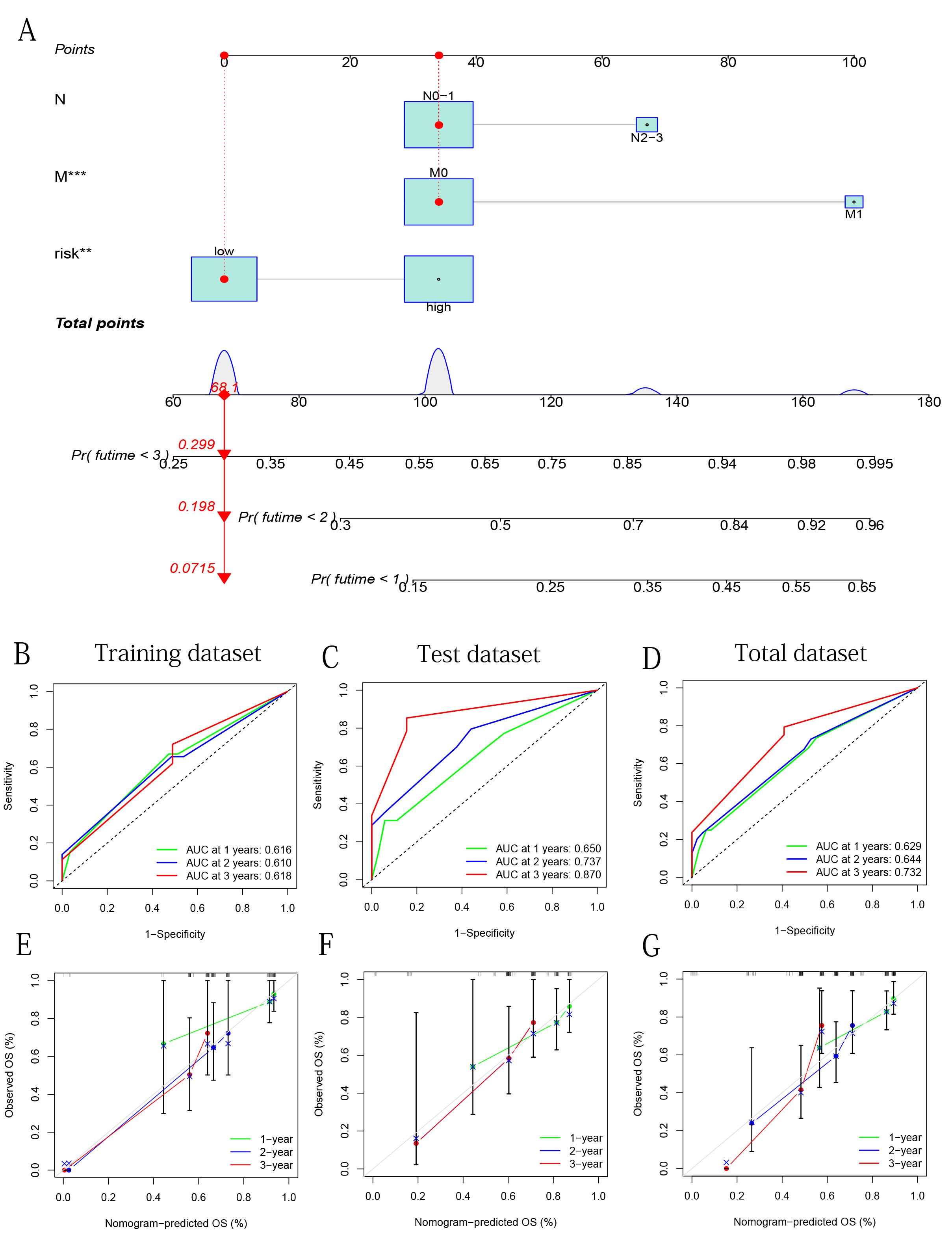

Supplement: Supplementary file 10 [file Image10.JPEG]

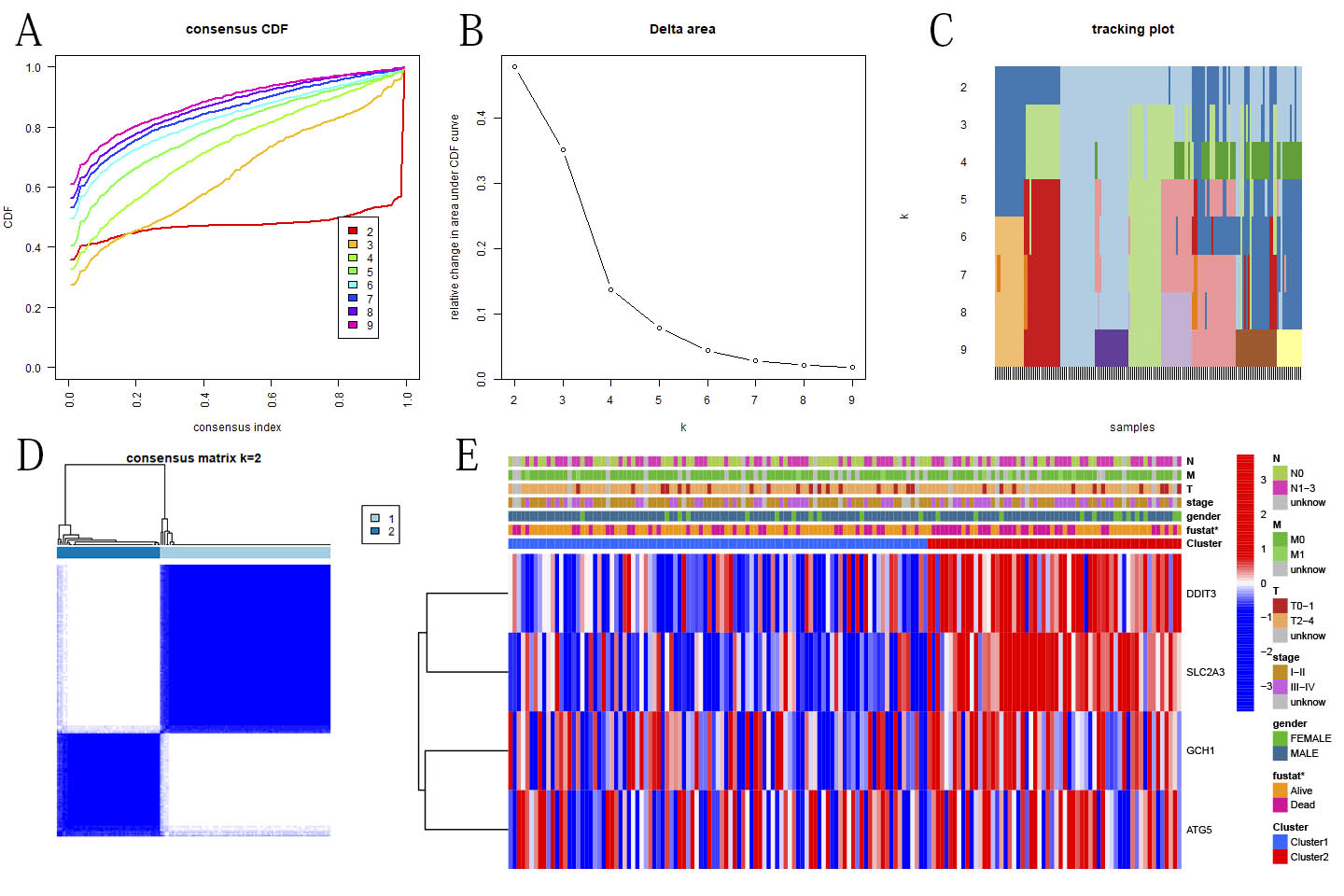

Supplement: Supplementary file 11 [file Image8.JPEG]

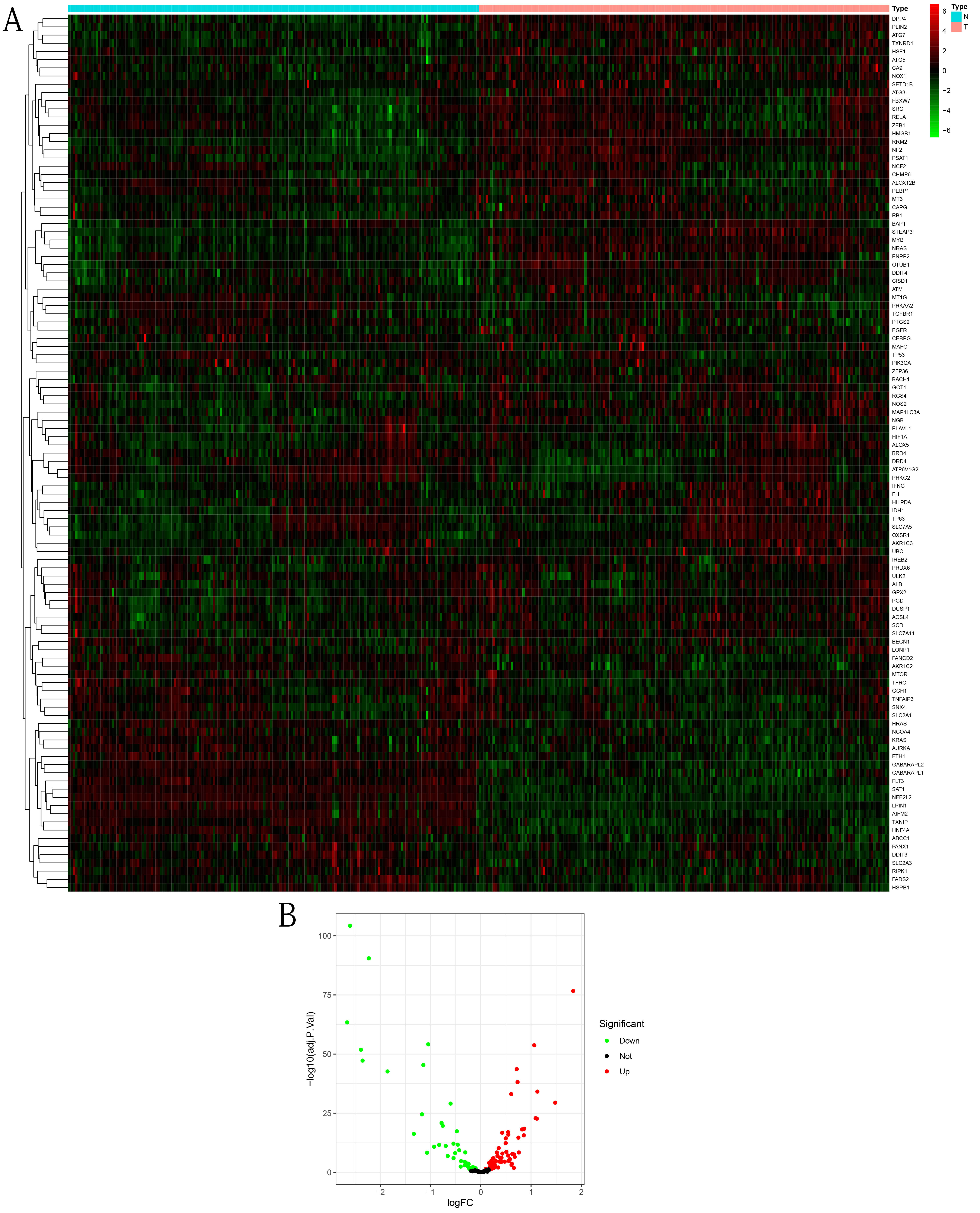

Supplement: Supplementary file 12 [file Image6.JPEG]
